# A framework genetic map for *Miscanthus sinensis *from RNAseq-based markers shows recent tetraploidy

**DOI:** 10.1186/1471-2164-13-142

**Published:** 2012-04-24

**Authors:** Kankshita Swaminathan, Won Byoung Chae, Therese Mitros, Kranthi Varala, Liang Xie, Adam Barling, Katarzyna Glowacka, Megan Hall, Stanislaw Jezowski, Ray Ming, Matthew Hudson, John A Juvik, Daniel S Rokhsar, Stephen P Moose

**Affiliations:** 1 Energy Biosciences Institute, Institute for Genomic Biology, University of Illinois Urbana, 1206 West Gregory Drive, Urbana, IL 61801, USA; 2 Crop Sciences, Edward R. Madigan Laboratory, University of Illinois, 1201 West Gregory Drive, Urbana, IL 61801, USA; 3 Energy Biosciences Institute, University of California, 130 Calvin Laboratory, Berkeley, CA 94720, USA; 4 Department of Molecular and Cell Biology, Life Sciences Annex, University of California, Berkeley, CA 94720, USA; 5 Institute of Plant Genetics, Polish Academy of Sciences, Strzeszynska 34, 60-479 Poznan, Poland; 6 Department of Plant Biology, Edward R. Madigan Laboratory, University of Illinois, 1201 West Gregory Drive, Urbana, IL 61801, USA; 7 DOE Joint Genome Institute, 2800 Mitchell Drive, Walnut Creek, CA 94598, USA; 8 National Institute of Horticultural & Herbal Science, Rural Development Administration, Suwon 440-706, Republic of Korea; 9 Present address: Department of Biology, Center for Genomics and Systems Biology, New York University, New York, NY, USA

## Abstract

**Background:**

*Miscanthus *(subtribe Saccharinae, tribe Andropogoneae, family Poaceae) is a genus of temperate perennial C4 grasses whose high biomass production makes it, along with its close relatives sugarcane and sorghum, attractive as a biofuel feedstock. The base chromosome number of *Miscanthus *(x = 19) is different from that of other Saccharinae and approximately twice that of the related *Sorghum bicolor *(x = 10), suggesting large-scale duplications may have occurred in recent ancestors of *Miscanthus*. Owing to the complexity of the *Miscanthus *genome and the complications of self-incompatibility, a complete genetic map with a high density of markers has not yet been developed.

**Results:**

We used deep transcriptome sequencing (RNAseq) from two *M. sinensis *accessions to define 1536 single nucleotide variants (SNVs) for a GoldenGate™ genotyping array, and found that simple sequence repeat (SSR) markers defined in sugarcane are often informative in *M. sinensis*. A total of 658 SNP and 210 SSR markers were validated via segregation in a full sibling F1 mapping population. Using 221 progeny from this mapping population, we constructed a genetic map for *M. sinensis *that resolves into 19 linkage groups, the haploid chromosome number expected from cytological evidence. Comparative genomic analysis documents a genome-wide duplication in *Miscanthus *relative to *Sorghum bicolor*, with subsequent insertional fusion of a pair of chromosomes. The utility of the map is confirmed by the identification of two paralogous C4-pyruvate, phosphate dikinase (C4-PPDK) loci in *Miscanthus*, at positions syntenic to the single orthologous gene in *Sorghum*.

**Conclusions:**

The genus *Miscanthus *experienced an ancestral tetraploidy and chromosome fusion prior to its diversification, but after its divergence from the closely related sugarcane clade. The recent timing of this tetraploidy complicates discovery and mapping of genetic markers for *Miscanthus *species, since alleles and fixed differences between paralogs are comparable. These difficulties can be overcome by careful analysis of segregation patterns in a mapping population and genotyping of doubled haploids. The genetic map for *Miscanthus *will be useful in biological discovery and breeding efforts to improve this emerging biofuel crop, and also provide a valuable resource for understanding genomic responses to tetraploidy and chromosome fusion.

## Background

The grass subtribe Saccharinae (sugarcanes, sorghums, miscanthus, and related C4 species) includes a remarkable array of recently and independently derived polyploids that arose from a common diploid progenitor. For example, sugarcanes carry even multiples of a haploid complement of x = 10 or x = 8 chromosomes, and exhibit polysomic inheritance that presumably arose via auto-polyploidy [1-3] over the past several million years. This scenario is consistent with the similar monoploid DNA content of sugarcane (approximately 750 million base pairs (Mbp) for *S. spontaneum, *930 Mbp for *S. officinarum *[4] and 730 Mbp for *Sorghum bicolor *[5]. The ten chromosome pairs of diploid *S. bicolor *likely represents the ancestral Saccharinae condition. Polyploidy in *Saccharum *arose at least twice, and chromosome number in sugarcane is so flexible as to allow a range of natural and artificial auto- and allo-polyploids up to dodecaploid.

In contrast, the genus *Miscanthus *has a base chromosome number of x = 19, with nominally diploid (2 N = 2x = 38) and tetraploid (2 N = 4x = 76) species, plus the highly productive triploid interspecific hybrid, *Miscanthus x giganteus*. Among a number of possibilities for the distinctive chromosome number, the most likely is the whole genome duplication (tetraploidization) of an ancestor possessing N = 10 pairs of chromosomes [6], although this has not been demonstrated. Direct comparisons of the DNA content of *Miscanthus *to sorghum and sugarcane is not obviously informative, as the N = 19 monoploid DNA content of *Miscanthus *spans 2150-2650 Mbp [7], more than three times longer than the monoploid content of eusorghum (745-818 Mbp) [8]. The possible origin of the nearly doubled chromosome number and tripled haploid size via polyploidy is further obscured by the high repetitive content of the *Miscanthus *genome, recently shown by sample sequencing to be ~95% in *M*. x *giganteus *[9].

Chromosome numbers can be unreliable indicators of even relatively recent polyploidy. For example, 2 N = 20 maize is a paleopolyploid comprising two sub-genomes that diverged ~12 Mya [10]. Comparative mapping and sequence analysis reveals that the progenitors of these sub-genomes also had 2 N = 20, a fact obscured karyotypically by subsequent chromosome fusions in the maize lineage. Conversely, while diploid *Sorghum bicolor *has 10 pairs of chromosomes, other diploid *Sorghum *species with comparable DNA content have only five pairs, presumably a consequence of chromosomal fusions [8]. Similarly, diploid *Brachypodium distachyon *has 2 N = 10 chromosomes, but other *Brachypodium *species with comparable DNA content have 2 N = 20 [11]. In any event, even in a whole-genome duplication scenario, the odd base chromosome number of *Miscanthus *would require additional chromosome-scale events such as loss or fusion. The description of *M. sinensis *as "diploid" with 2N = 38 chromosomes is based on chromosome counting, and the observations that chromosome pairing during meiosis regularly produces bivalents [12,13].

Despite *Miscanthus*' unusual chromosome and DNA complement relative to other Saccharinae, relatively few genetic resources have been developed for elucidating the relationship of the *Miscanthus *genome to those of its close relatives. This is in part due to the fact that the most widely grown *Miscanthus *biomass crop is the vegetatively propagated triploid *M*. x *giganteus *(3x = 57), which produces no viable seed [14], and therefore no segregating progeny. *M*. x *giganteus *is among the most productive known grasses [15] and evidence to date indicates it derives from a cross between a diploid *M. sinensis *father and a tetraploid *M.sacchariflorus *mother [16]. Another complicating factor is self-incompatibility, which makes the production of homozygous genotypes difficult and forces the independent mapping of meiotic products from each parent in F1 progeny.

*M. sinensis*, the likely diploid parent of *M*. x *giganteus *[16], is widely grown as an ornamental grass with rich genetic diversity, and is itself highly productive. Although a preliminary genetic linkage map for *M. sinensis *using RAPD markers and an "offspring cross" mapping strategy has been published [17], this map resolves 28 linkage groups (LGs), many more than the expected 19 LGs. The marker density of the map is not sufficient for fine-scale mapping and the reproducibility of RAPD markers is difficult. These problems can be mitigated by utilization of simple sequence repeat (SSR) and single nucleotide polymorphism (SNP) markers, which are plentiful in the *Miscanthus *genome and are also reproducible across laboratories. Additionally, SSR markers can be used for the search of homoeologous chromosomes in the mapping of polyploid plants [18,19].

Here we report the discovery of genetic variation in *Miscanthus sinensis *using SNP markers discovered by both deep transcriptome sequencing and amplification of SSRs that were previously shown to be variable in sugarcane. Analysis of the segregation of these variants in a reciprocal F1 cross, as well as genotyping two doubled haploids and their diploid parents, reveals both allelic (segregating) and widespread paralogous (fixed) sequence differences. We obtained a dense map of all 19 linkage groups in *M. sinensis *with 846 segregating markers. Comparison with the *Sorghum bicolor *genome reveals a whole-genome duplication in *Miscanthus*, with a single chromosome fusion accounting for the odd base chromosome number of the genus. The two sub-genomes of *Miscanthus *are quite similar, resulting in variant frequencies among paralogs that are only modestly higher than those observed between alleles. Despite this recent duplication, whether by allo- or auto-tetraploidy, our map is consistent with disomic inheritance in *Miscanthus*, in contrast to the polysomic inheritance found in the closely related polyploid sugarcane. Our genetic map of *Miscanthus *provides a valuable resource that can be used to apply both functional genomics to this perennial C4 grass, and marker-assisted breeding to biomass crop improvement.

## Methods

### Grosse Fontaine x Undine reciprocal mapping population

**Figure 1:**
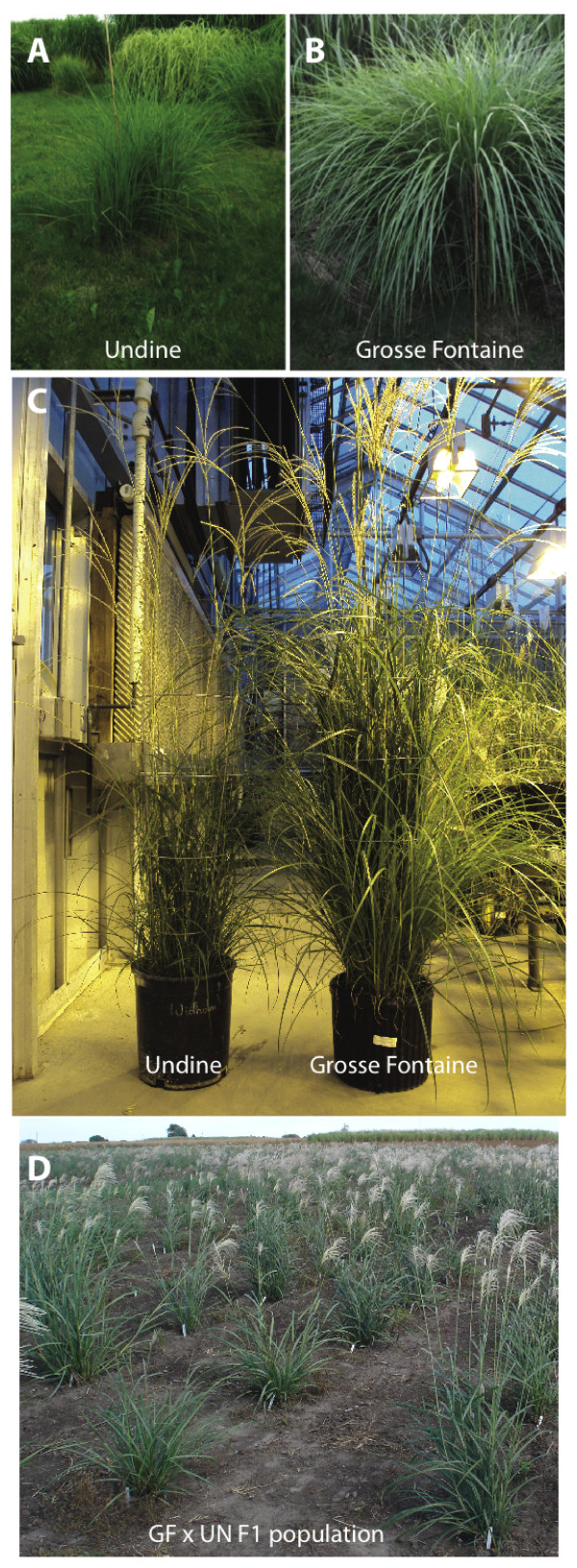
**Parents of the *M. sinensis *mapping population**. A full sib (F1) population was produced by reciprocally crossing two ornamental *M. sinensis *accessions, "Grosse Fontaine" (GF) and "Undine" (UN). The parents of the mapping population were propagated from rhizomes taken from two individual plants, UN (panel A, plant location SF20) and GF (panel B, plant location SF5), established by Emily Heaton in the spring of 2001 at the SoyFACE plot located at the Crop Sciences Research and Education center, on the University of Illinois campus. The photographs of GF and UN were taken on July 11 2011. Panel B shows the individual GF and UN plants used to make the F1 mapping population, flowering in the greenhouse (photograph taken on Dec 14th 2009). Phenotypic measurements reported in Additional file 1: Table S1, were taken on these two plants at maturity. Panel C shows the full sib F1 mapping population in August 2010 at the University of Illinois Energy Farm. This population was established in the spring of 2010.

A full sib (F1) population was produced by reciprocally crossing two ornamental *M. sinensis *accessions, 'Grosse Fontaine' (GF) and 'Undine' (UN) propagated in the greenhouse (Figure 1C), from rhizomes of single plants established at the SoyFACE plot (Figure 1A and 1B; plant locations SF20 and SF5 respectively) located at the Crop Sciences Research and Education center, on the University of Illinois campus. The temperature of the greenhouse was maintained between 22.2-29.4°C and supplemental light (threshold of 600 W/m2) was provided from 6 am to 8 pm. These accessions are variable for a number of phenotypes (Additional file 1: Table S1). All measurements reported were taken from mature plants growing in the greenhouse (Figure 1C), including plant height, flowering time and leaf shape. Parent plants were isolated in a greenhouse room and reciprocally cross-pollinated to produce seeds from both parents. Seeds were collected separately for each direction of the cross and germinated in seed trays in the Plant Science Laboratory greenhouse at the University of Illinois at Urbana-Champaign (UIUC). A full -sib population from both reciprocal crosses was grown in the greenhouse. The rhizome from each individual was split and planted in a randomized block design with 3 clonal replicates per plant at the Energy Biosciences Institute (EBI) farm at UIUC in May 2010 (Figure 1D).

Genomic DNA of the mapping population and the two parental genotypes was extracted from young leaves using the Puregene protocol (Qiagen, Valencia, California, USA) and used for SSR and SNP marker development and genotyping. After removing individuals that showed non-parental alleles, likely due to pollen contamination, 221 F1 individuals defined our mapping population, including 113 with GF as maternal parent and 108 with UN as the maternal parent. All plants were genotyped for mapping using SNP and SSR markers as described below.

### Transcriptome sequencing and assembly

Total RNA was extracted from young leaves from GF and UN (the two parents of the mapping population) using a CTAB RNA extraction method [20]. Paired-end RNA-seq libraries were made using the Illumina RNAseq kit (cat # RS-930-1001) as per the manufacturer's instructions. The libraries were sequenced at the Keck Center for Functional Genomics at the University of Illinois on an Illumina GA II platform. A total of 144 million 80 bp RNAseq reads were generated from 6 lanes of sequencing, with 5 of the lanes producing successful paired-end reads (found at NCBI short read archive, accession number SRA051293).

*De novo *assembly of the raw RNAseq reads for each parent was performed using ABySS [21] with k-mer lengths k = 25, 30, 35, 40, 45 and 50 bp. All assemblies were run on fifteen nodes of a cluster (Dual-quad cores (2.83 GHz Xeons), 16 GB RAM). The assemblies were made non-redundant by removing contigs that were identical or completely contained within a larger contig. The resulting contigs from Undine and Grosse Fontaine were then merged using Phrap (Green et al., unpublished observations) version 1.080721, -revise_greedy, -minmatch = 20 and -penalty = -9). This combined assembly (Additional file 2), was used as the reference sequence for the discovery of single nucleotide variants (SNVs).

### Identification of single nucleotide variations from RNAseq data

RNA-seq reads were aligned back to the combined Undine and Grosse Fontaine transcriptome assembly using Bowtie [22,23] and bwa [24,25]. Bowtie was run with the -k option set to 1 and with the -best option. Bwa was run with -q 15. The sam output was converted to bam and sorted using view and sort functions from the samtools suite [26]. Duplicate reads were removed using the samtools rmdup function as these could be an artifact of the PCR step during the construction of the RNAseq libraries. The bam file was then converted to pileup format using samtool's pileup function and SNVs were identified computationally using VarScan [27]. For the GoldenGate probe set, only SNVs flanked by at least 50 bp of invariant sequence that had a minimum of ten reads corroborating each allele were chosen. There was no tolerance for indels.

To obtain probes appropriate for genotyping with genomic DNA, we screened these 101 bp sequences (the SNVs chosen for the GoldenGate assay plus the 50 invariant bases on both flanks) using BLAT [28] against the fully assembled genomes of four grasses (sorghum, maize, rice, and *Brachypodium distachyon*) to eliminate sequences that contained splice junctions. Illumina further filtered probes for robustness with respect to the GoldenGate assay. Additional file 3: Table S3, contains the final SNV set and assay details required to order the array.

### Single nucleotide variant (SNV) genotyping using the GoldenGate™ and Genome Studio

Genomic DNA from the F1 mapping population and both parents, as well as two doubled haploid *M. sinensis *(IGR-2011-001 and IGR-2011-002) and their parents (IGR-2011-003 and IGR-2011-004, respectively), were assayed at the Keck Center for Functional Genomics at the University of Illinois using the 1536 SNV GoldenGate array described above, following the manufacturer's protocols. Genotypes were called using Genome Studio (Illumina), which characterizes each genotype according to the signal intensities measured for the alternate nucleotides that define a SNV. Here and below we denote these alternate nucleotides "A" and "B."

**Figure 2:**
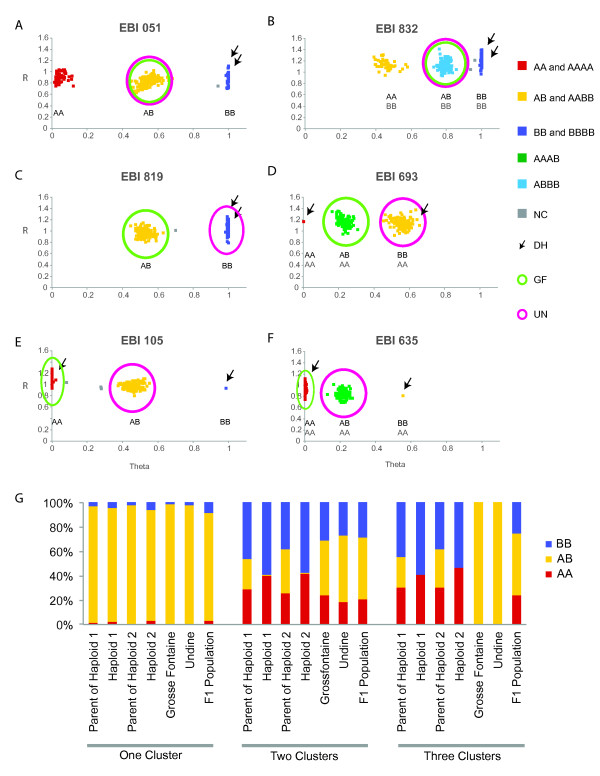
**Genotype calling using the *Miscanthus *GoldenGate™ array**. The graphs in panels A-F plot normalized theta (ratio of signal intensities assayed for A and B SNP alleles) against normalized R (signal intensity) for each individual represented as a colored square. Panels A, C, and E illustrate markers that cluster as predicted for a biallelic SNP, which segregate as AA (red), AB (yellow), or BB (blue). Panels B, D, and F illustrate markers that cluster as predicted for a SNP distinguishing alleles for one of two duplicated and unlinked loci, where theta is skewed by the relative dosage of A and B SNPs. In all panels, clusters are defined as sharing alleles with either the Grosse Fontaine (green circles) or Undine (pink circles) parents, individuals that fall outside the cluster are marked as "no calls" (NC, grey), and the doubled haploid genotype is indicated by the black arrow. Panel G reports the relative fraction of genotyped segregating SNPs within each clustering type among the Grosse Fontaine and Undine parents, the population of their F1 progeny, as well as the two doubled haploids and their respective parents. Single cluster markers (fixed differences between paralogs) behave similarly in diploids and doubled haploids. In contrast, while diploid accessions show extensive heterozygosity at segregating loci (two- and three-cluster markers), doubled haploids show no heterozygosity.

For each SNV, Genome Studio clusters signal intensities to define homozygous and heterozygous genotype calls. For a segregating (diploid) SNP, one or two homozygous clusters and one heterozygous cluster are expected, depending on whether or not the SNP is variable in both parents (Figure 2A, C and 2E show clusters observed in biallelic SNPs). In contrast, a SNV that represents a fixed single nucleotide difference between paralogs (*i.e*., a non-segregating variant at two unlinked loci) is revealed when both parents, and the entire F1 population, form a single "heterozygous" cluster (data not shown). SNVs that are fixed at one locus but segregating at another form two or three clusters as for a conventional diploid SNP, but with skewed signal intensities (Figure 2B, D and 2F). Each SNV cluster was reviewed manually in Genome Studio. SNVs forming more than three clusters were discarded. Two doubled haploid lines and their parents were also genotyped, and used to confirm clustering (Figure 2).

### Simple sequence repeat (SSR) marker development

Primers for sugarcane SSRs derived from expressed sequence tags (ESTs) and intergenic sequences were previously designed and characterized by James et al., 2011 [29]. We tested these primers in *M. sinensis *to screen for markers that are polymorphic within one or both parental genotypes, GF and UN. Products were amplified in 10 μl PCR reactions containing 1 μl of genomic DNA (5-10 ng) from GF or UN, 0.1 μl of forward and reverse primers (100 μM stock each, Additional file 4: Table S4), 3.8 μl of ddH2O and 5 μl of 2X GoTaq Green Master Mix (Promega, Madison, Wisconsin, USA). PCR conditions for the screening were as follows: 3 min of denaturation at 94°C, 36 cycles of 94°C for 30 sec, 55°C for 30 sec and 72°C for 45 sec followed by a final extension at 72°C for 10 min. The amplicons were separated on 4% agarose SFR gels (Amresco, Solon, Ohio, USA) with 1X TBE buffer at 4°C and visualized with ethidium bromide. Polymorphic markers resulting from this screen were used for subsequent genotyping of the *Miscanthus *mapping population (Additional file 5: Figure S2).

To genotype the mapping population, products were amplified in 10 μl PCR reactions containing 1 μl of genomic DNA (5-10 ng), 0.02 μl of M13 tailed forward primer, 0.1 μl of each reverse and fluorescent M13 primers (100 μM stock), 3.78 μl of ddH2O and 5 μl of 2X GoTaq Colorless Master Mix (Promega, Madison, Wisconsin, USA). Four M13 primers tagged with FAM, VIC, NED and PET at the 5' end were used in this analysis to fluorescently label the SSR amplicons. All primers were ordered from Integrated DNA Technologies (idtdna.com). Touchdown PCR was used to amplify the SSRs: denaturation at 94°C for 3 min followed by 2 cycles of 94°C for 30 sec, 65°C for 30 sec, and 72°C for 45 sec. The annealing temperature was decreased every 2 cycles by 2°C until 57°C. The amplification was finished with 26 cycles of 94°C for 30 sec, 55°C for 30 sec, and 72°C for 45 sec (total 36 cycles) and a final extension at 72°C for 10 min. Electrophoresis of the amplicons was carried out by the Keck Center of functional genomics at the University of Illinois, on an ABI 3730*xl *with the LIZ600 size markers. Marker scoring was done using the Genemarker software (Softgenetics, LLC State College, Pennsylvania, USA).

### Linkage analysis and map construction

The 221 F1 offspring were genotyped using fragment analyses of 210 amplicons from 107 SSR primers and GoldenGate analysis of 1536 SNVs (Table 1). A total of 868 markers showed clear polymorphisms or clustering and were used in the linkage analysis. Genotypes were converted into JoinMap "CP" (cross pollinator) codes based on the genotypes of the parents and the type of marker. The linkage map was constructed using the JoinMap 4.1 software [30]. Thirty-five of the markers displayed a segregation ratio greater than 2:1 when the expected ratio was 1:1, and greater than 10:1 when the expected ratio was 3:1 (Table 1). These 35 markers were not considered during the construction of the initial framework map. None of the individuals included had missing data for more than 13 markers. A minimum independence logarithm of odds (LOD) score of 11 and a maximum recombination frequency of 0.4 was used to define linkage groups in all map calculations mentioned here on.

**Table 1:** Summary of SSR and SNP Marker

Type of Marker	Number of primer pairs/SNPs	Number of amplicons	Polymorphic amplicons	Markers polymorphic in Undine		Markers polymorphic in Grosse Fontaine		Markers polymorphic in both parents		
				**Segregation ratio**						

				1:1	> 2:1	1:1	> 2:1	3:1	> 10:1	1:2:1

EST SSRs	29	91	73	33		21	2	15	2	

Genomic SSRs	78	210	137	48	2	50		25	12	

SNPs	1536	n/a	n/a	238	16	212	1			191

Total		n/a	n/a	318	17	280	0	40	0	191

### Synteny with *Sorghum bicolor *genome

Mapped *Miscanthus *markers were aligned to the *Sorghum bicolor *genome using blastn 2.2.25+ [31] with wordsize 10 and BLAT [28] (default parameters). From these two alignments the SNP markers were assigned to a position in sorghum if they had the largest number of identical residues and shared at least 80% of the residues in the probe. The positions of markers in centiMorgans on the 19 *Miscanthus *linkage groups were plotted versus these aligned positions to the sorghum genome coordinates.

### Comparison to sorghum genetic map

The consensus map for *Sorghum bicolor *developed in Mace et al. [32] was adopted. Sequence-tagged markers were extracted from supplemental materials of this paper and Genbank, and aligned to the chromosome sequences of sorghum [5]. Sorghum map positions for our *Miscanthus *markers were then inferred by linear interpolation using flanking markers from the sorghum map, assuming locally constant recombination rates.

### Sequence of the 3rd intron of *Miscanthus *C4-PPDK

PPDK sequences in Genbank (accession numbers AY262272.1, AY262273.1), and the GF and UN RNAseq sequences and assemblies were aligned to the genomic PPDK locus on sorghum chromosome 9. Primers PPDK-int3F and PPDK-int3R (5'-AACCTGGCGGAGATGTCGA-3' and 5'-AGGTAGACTTCCTTGTACTGA-3' respectively) were designed to amplify the third intron of C4-PPDK from both Undine and Grosse Fontaine. The primers amplified two fragments, between 1500 and 2000 bp, from each parent. Each amplicon was cloned separately into pGEM-t easy (Promega) and a total of 45 clones (10 to 15 clones from each band) were Sanger sequenced using three oligonucleotide primers, (SP6, T7 and 5'-GAGACAGCGATTGGACTAAGC-3'). The sequences were aligned using the Sequencher sequence analysis software (Gene Codes Corporation, Ann Arbor, MI USA).

### Phylogenetic analysis of intron sequences

Intron and flanking exon sequences from primer to primer were aligned with Muscle [33] and trimmed to remove ambiguous sites. Orthologous introns from *S. bicolor, S. officinarum*, and *Z. mays *were identified from sequences in Genbank. For the purposes of phylogenetic analysis, identical sequences were removed. Gblocks [34] was used to identify blocks of well-aligned sequence with a minimum of 6 sequences for a conserved position, 8 for a flanking position, 8 as the maximum number of contiguous non-conserved positions, half allowed gap positions, and a minimum block size of 5. The final alignment had 1,337 positions. MrBayes [35] was used to produce a consensus phylogenetic tree (50,000 generations, with sampling frequency, 100), using an inverted gamma distribution for rate variation. Midpoint rooting was used.

### Mapping *Miscanthus *C4-PPDK loci

The G/A polymorphism at position 397 in the sequence alignment, shown in Additional file 6: Figure S6A, was used as a CAPS marker [marker identifier EBI-847] as this polymorphism results in the presence of an *Nhe*I restriction enzyme site (5'-GCTAGC-3'). *Nhe*I (NEB # R0131S) was used to digest amplicons obtained from PPDK-int3F and PPDK-int3R in the parents and population. The population was scored for the presence of one or two bands as marker EBI 847.

A second SSLP marker (EBI-848) was designed around two indels between positions 1354 and 1388. Oligos PPDK-UD3F and PPDK-UD3R (5'-AAAGGTGAACATAGTTTCG-3' and 5'-CATAGTTCG(T/A)AGCGTGAG-3' respectively), were designed around these indels (Additional file 6: Figure S6B) and used to amplify the locus from the population and the parents. The plants either amplified a single fragment (132 bp) or amplified two fragments (132 bp and 118 bp). The 118 bp amplicon segregated in the population and was scored as EBI 848.

## Results and discussion

### RNAseq and the genomic sequence of related species can be used to define SNVs

To develop a collection of putative SNVs for *Miscanthus*, we sequenced transcriptomes of *M. sinensis *'Grosse Fontaine' and 'Undine' leaves and leaf rolls using deep RNAseq. Across both accessions, we generated over 21 Gbp in predominantly paired 80 bp Illumina GA II reads (Additional file 7: Table S2, NCBI Short Read Archive accession number SRA051293). From these RNAseq data we assembled a unified set of 29,933 contigs longer than 100 bp (Additional file 2). The median contig length was 522 bp, with half of the total contig length accounted for by 6,433 contigs longer than 1,071 bp (the contig N50). We identified SNVs by realigning the RNAseq reads against the assembled transcriptome contigs and requiring strong support for two alternate variants embedded in otherwise nearly identical flanking sequence, to enable straightforward high-throughput genotyping. Other variation observable in the dataset was not considered further.

Since our aim was to define variants that could be genotyped by a GoldenGate assay with genomic rather that transcriptomic samples, we excluded from consideration probe sequences that spanned a putative exon-exon boundary. To do this in the absence of a *Miscanthus *genomic reference, we took advantage of the extensive conservation of exon-exon boundaries in grasses [5] to identify and reject likely exon-junction-spanning probe sequences by comparison with the genomes of sorghum, maize, and rice. To facilitate syntenic comparisons between *Miscanthus *and related species, we also chose for genotyping those SNVs that (1) could be readily assigned to homologs in sorghum by sequence similarity and (2) had homologs that were distributed across all sorghum chromosomes (Additional file 8: Figure S1).

### Results of GoldenGate genotyping

Out of 1,536 putative markers on the *Miscanthus *GoldenGate array (Additional file 3: Table S3), 1,243 showed one or more clusters in GoldenGate signal space (Figure 2), indicating consistent genotyping across individuals. The remaining 293 putative markers showed dispersed or very low signal in Genome Studio and were considered failed assays, and not investigated further. Of the 1,243 successful oligonucleotide assays, we found that 93 assays showed signal for only one probe, and appear to be homozygous across both parents and their progeny or represent cases where the second oligo probe failed. After excluding these failed or invariant assays, we were left with 1,150 markers, of which 658 formed 2 or 3 clusters in signal space. The remaining markers appear as either a single centrally located cluster, more than three clusters, or dispersed signal, and were not considered further.

### Intepretation of GoldenGate SNP genotypes

By considering the patterns of genotypes across our F1 mapping population, we found that many of the SNV's discovered by RNAseq analysis are indeed segregating biallelic markers (*i.e*., single nucleotide polymorphisms, or SNPs). Others, however, represent fixed differences between closely related paralogous loci. Furthermore, many segregating biallelic markers have their GoldenGate signal affected by a closely related paralog that has the same sequence as the marker allele. Signal from such paralogous alleles causes the cluster positions in Genome Studio to be skewed in a characteristic manner that is readily recognized. A plot of normalized theta (ratio of signal intensities assayed for A and B SNP alleles) against normalized R (signal intensity) per marker for each individual can be used to visualize genotypes in a segregating population (Figure 2A-2F). The values of normalized theta are close to 0 in samples where the genotype is AA, close to 0.5 if it is AB and close to 1 if it is BB.

In situations where more than one locus is being sampled, and where the sequence of a second (paralogous) locus matches one of the two allelic states of the SNV in the segregating locus, the clusters are skewed towards the allele sharing the common nucleotide (Figure 2B, C and 2D). In Figure 2B, locus 1 is heterozygous for A and B SNVs in both parents and hence produces AA, AB, or BB progeny, whereas the second paralogous locus is fixed for the B SNV in both parents and progeny. This results in all three clusters being skewed to the right due to the higher dosage of SNV B. Figure 2D shows a scenario where the GF parent is AB and the Undine parent BB at locus 1, whereas the second locus is fixed for SNV A in both parents and progeny, which shifts clusters to the left due to higher dosage of SNV A. A similar situation is shown in Figure 2F where UN rather than GF is segregating at locus 1. For mapping of segregating loci, panels A and B indicate markers that are heterozygous in both GF and UN parents, panels C and D show markers heterozygous in only the GF parent, and panels E and F markers heterozygous in only the Undine parent. Markers shown in Figure 2E and 2F share the feature where the genotype of the two different sampled doubled haploid lines carry either the A or B SNV, but no progeny share the B/B genotype because their parents have either an A/A (GF) or A/B (UN) genotype.

Notably, 26% of the two-cluster SNV's showed skewed signal intensities in the GoldenGate assay, indicating that the two alternative sequences are not present in equal dosages. This observation is consistent with the sequence variants being detected from more than one locus, and suggests that many of the variant sequence pairs A and B appear as heterozygous alleles at one locus (A/B) but are fixed at a second locus (*i.e.*, A/A or B/B), resulting in a ~3:1 ratio of signal intensities on the GoldenGate assay. If both parents show allelic variation at one locus but are fixed for the same allele at a second paralogous locus, then segregating progeny may show 2:2, 3:1, and 4:0 dosages, consistent with observations (Figure 2A. EBI 832, EBI 693 and EBI 635).

A second class of SNV (33%) formed only a single cluster of genotypes (data not shown). For these SNVs, both parents and all progeny had the same genotype. This is consistent with the pattern expected from fixed differences between paralogous loci (e.g., A/A at one locus and B/B at another) that do not segregate in progeny. These SNV's are not useful as genetic markers, since both parents and all progeny fall into a single "heterozygous" cluster and there is no genetic segregation of alleles. The proportion of both single cluster and skewed two-cluster SNVs (59%) should not be used as a direct estimate of the degree of paralogy due to the potential biases introduced by our SNV discovery and selection. These paralogous loci, however, do suggest extensive paralogy in the *Miscanthus *genome, which is corroborated by the genetic map as shown below.

Only a small minority (5 out of 1536) of the SNVs that we identified by RNAseq analysis formed more than three clusters in signal space, and could not be simply interpreted either as segregating alleles or fixed paralogous variants. The rarity of such SNV's in this analysis suggests that a similar RNAseq-based protocol could be useful in SNP discovery from other *Miscanthus *populations and species lacking genomic reference sequences.

For 658 out of 1,150 genotyped *Miscanthus *SNVs, the GoldenGate intensities in our F1 mapping population could be grouped into two (467) or three (191) clusters of genotypes in signal space, indicating variants that are found in both homozygous and heterozygous states in the population. We interpreted the two-cluster class of SNV's as segregating SNPs that are heterozygous in one parent and homozygous in the other, with progeny of both types. Similarly, the three-cluster classes of SNVs are interpreted as SNPs that are heterozygous in both parents, allowing for homozygous offspring of two types as well as heterozygotes. The interpretation of these SNV as segregating SNPs in our cross is supported by the integration of these markers into a consistent linkage map with limited segregation distortion (below).

### Corroboration of allelic and fixed differences using doubled haploid lines

To test our hypothesis that many SNV's represent fixed differences between paralogous loci, we also genotyped two *M. sinensis *double haploid lines and their parents. Since the doubled haploids were developed by another culture from outbred diploid parents (Glowacka, unpublished observations), we had two expectations.

First, for the SNV's that are inferred to be biallelic SNPs in our F1 cross, we expect that some of them will correspond to heterozygous loci in other *M. sinensis *accessions, including the outbred parents of the doubled haploid lines. If these SNV's are *bona fide *allelic variants, however, then the doubled haploids should be homozygous for all such variants. Figure 2G shows the segregation of alleles in the GoldenGate assay. In situations where two or three clusters are observed in the GoldenGate, consistent with a biallelic SNP, the double haploids are either A/A or B/B homozygotes while the mapping population has all three allelic states, as expected.

Second, for SNV's that are inferred to be fixed differences between paralogs, both variant states should be observed in the doubled haploids as well as their parents. This is observed as a single AB cluster on the GoldenGate array (Figure 2G).

### Genotyping summary

Taken together, our analyses of the F1 mapping population and the two doubled haploid lines show that we can distinguish segregating allelic variants at a single locus from fixed differences between paralogs, even in the face of extensive gene duplication. These data suggest that many *Miscanthus *genes have a closely related paralog that cannot be easily differentiated in the short read transcript data, but which assort independently. Using segregation patterns from a high density of genetic markers a linkage map can be constructed.

### SSR primers from sugarcane identify allelic and paralogous polymorphism in *Miscanthus*

Since Saccharum (sugarcane) is a close relative of *Miscanthus*, we reasoned that primer pairs that amplify simple sequence repeats in *Saccharum *would also be likely to amplify polymorphic SSRs in *Miscanthus *[36]. Sixty-eight percent of the 2,640 SSRs primer pairs mined from sugarcane ESTs produced amplicons when tested with *Miscanthus*. Only 51% of the 2,628 SSR primer pairs derived from *Saccharum *genomic sequences produced amplicons with *Miscanthus*. Of these, 188 EST- and 237 genome-derived primers generated polymorphic amplicons between the two parental genotypes. Primers that produced non-specific amplicons were excluded. We genotyped the F1 mapping population using 107 primers pairs (29 and 78 primers from EST and intergenic sequences, respectively) out of 425 polymorphic primers. One hundred and seven primers produced 20 marker configurations (Additional file 4: Table S4, Additional file 5: Figure S2). Among them, 69 primers follow disomic marker configurations but 38 primers (35.5%) do not fit disomic configurations, producing more than 3 amplicons in one or both parents (Additional file 4: Table S4). One hundred and seven primers produced a total of 301 amplicons and among them, 210 were polymorphic between two parental genotypes and segregated in progeny populations. One hundred ninety three amplicons out of 210 were actually mapped (Table 1 Additional file 9: Table S5).

### An integrated linkage map for *M. sinensis*

Using the 868 segregating markers defined above, we constructed an integrated linkage map for *M. sinensis *using JoinMap 4.1. We took advantage of a newly implemented multipoint maximum likelihood model for constructing a map from an F_1 _cross of two outbred parents, using the Haldane mapping function [30]. In contrast to a pseudo-testcross approach, which utilizes markers that are heterozygous in one parent but homozygous in the other, the new method can also incorporate markers that are heterozygous in both parents. While pseudo-testcross based analysis results in separate maps for each parent, the combined approach allows direct integration into a single map of crossovers that occur in either or both parents by using the markers that are heterozygous in both parents as anchors.

**Figure 3:**
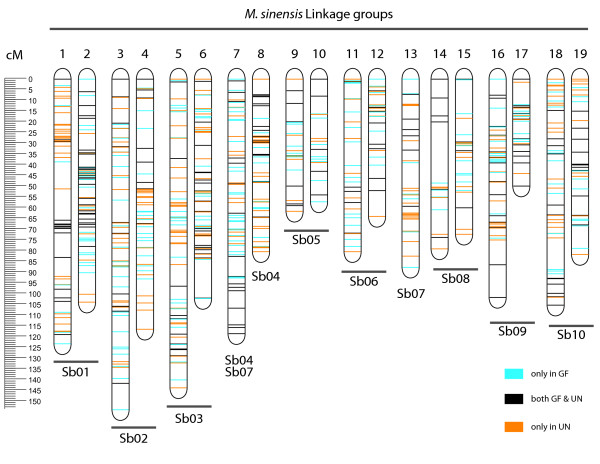
**Integrated genetic map for *M. sinensis***. Markers on each of the 19 linkage groups are shown as horizontal lines at their estimated map position on the integrated map, with scale in centiMorgans on left. Marker type is shown by color (blue, heterozygous in GF only; orange, heterozygous in UN; black, heterozygous in both GF and UN). Markers that are heterozygous in both parents allow individual parental maps to be combined. Marker types and positions are specified in Additional file 9: Table S5. Linkage groups are numbered based on extensive synteny with sorghum (see Figure 4).

Only 48 out of 868 markers show segregation distortion (p < = 0.005 using the chi-squared goodness of fit test). Of these, 35 were highly distorted markers, and not included in the initial framework map. These highly distorted markers include 21 with a segregation ratio greater than 2:1 when they should have been 1:1, and 14 with a segregation ratio greater than 10:1 when they should have been 3:1 (Table 1). Of the remaining 833 segregating markers (641 SNPs and 192 SSRs), 829 were incorporated into 19 major linkage groups using a minimum logarithm of odds (LOD) score of 11 and maximum recombination frequency of 0.4. The JoinMap 4.1 maximum likelihood method was used to calculate the map order of the framework map. Four SNP markers that were placed more than 40 cM away from the nearest marker on the linkage group were excluded and marker order for those linkage groups was recalculated. An attempt was then made to replace the 35 highly distorted markers on the ML map, keeping the marker order of the framework map constant and using the same map calculation parameters as before. Seventeen of the 35 highly distorted markers were incorporated. This map with 846 markers is shown in Figure 3.

Independent regression maps for each parent were also constructed to corroborate the robustness of marker order (Additional file 9: Table S5, Additional file 10: Figure S3 and Additional file 11: Figure S4). The total length of the 19 linkage groups on the ML map is 1782 cM, with an average intermarker spacing of 2.7 cM (excluding markers with identical map positions). Thus we expect that the missing map length from the telomeric ends of the linkage groups [37,38] accounts for roughly 2 × 19 × 2.7 cM = 102 cM, for a total estimated map length of 1884 cM. In the Grosse Fontaine map, 94% of the markers lie within 10 cM of each other, while in the Undine map only 90% meet this criterion. In the integrated map, 97% of the mapped markers lie within 10 cM of another marker, attesting to the dense coverage of the map.

### Disomic inheritance and limited segregation distortion

Transmission of each linkage group is consistent with pure disomic inheritance in *M. sinensi*s (*i.e*., complete preferential pairing of homologs), with no evidence for tetrasomic inheritance (*i.e.*, pairing and recombination between homoeologs). Furthermore, very few markers show segregation distortion (48 out of 868; p < = 0.005 using the chi-squared goodness of fit test), and those that do are concentrated on Ms2, Ms3, Ms4, Ms12, and Ms13. Overall there is more segregation distortion in Undine. Twenty of the 24 distorted UN markers lie on Ms4 (Additional file 12: Table S6). Potential causes of segregation distortion include the following three possibilities: (1) Failure to complement deleterious recessive alleles heterozygous in both GF and UN parents that reduce viability of F1 progeny; (2) Interactions between genomes, e.g., meiotic drive in F1 gametophytes, gametophytic competition or pollen-pistil interactions like self-incompatibility; (3) Proximity to areas of suppressed recombination like centromeres and nucleolus organizer regions. The design of our cross makes it difficult to differentiate among these possible explanations.

### Whole genome duplication with extensive conserved synteny to sorghum

Since our *Miscanthus *markers were derived from (1) transcribed regions with reduced sequence variation (SNPs) and (2) sequences from conserved ESTs and intergenic regions (SSRs), many of them could be unambiguously assigned to orthologous (*i.e.*, evolutionarily homologous) positions on the *Sorghum bicolor *genome sequence by straightforward sequence alignment. Out of 653 SNP loci on the integrated *Miscanthus *map, 618 could be placed on the sorghum genome. Similarly, out of 193 SSRs on the map, 126 could be placed on the sorghum genome.

**Figure 4:**
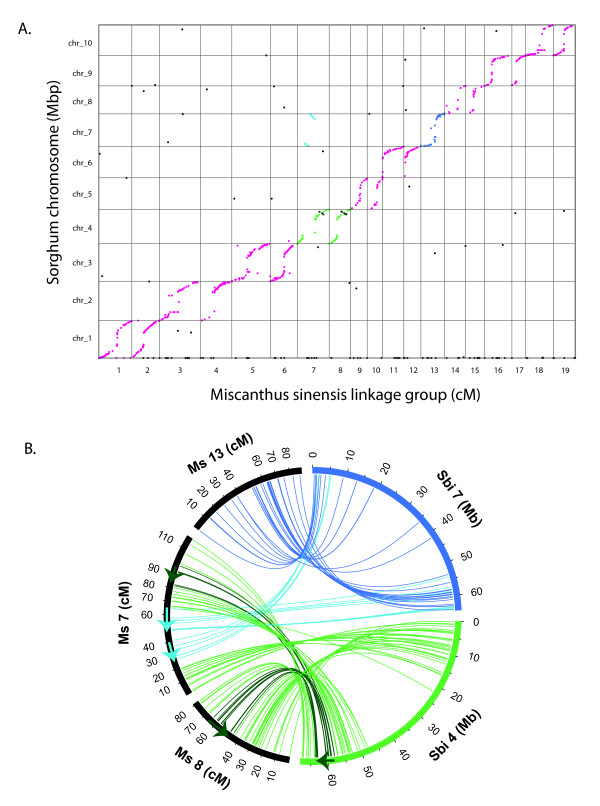
**Tetraploidy of *Miscanthus *relative to sorghum, with extensive colinearity and a single chromosome fusion**. **Panel A**. Horizontal axis shows genetic map position of markers on the 19 *Miscanthus *linkage groups, in centiMorgans; vertical axis shows physical map position of markers aligned to the 10 sorghum chromosomes in megabases. Each dot corresponds to a single marker. Markers that could not be uniquely mapped to sorghum are shown along the horizontal axis as black dots. Duplication and colinearity of nearly all chromosomes is evident (markers in magenta). A copy of sorghum chromosome 7 (markers in sky blue) has been inserted into a copy of sorghum chromosome 4 (markers in green) to produce *Miscanthus *linkage group 7. Markers on *Miscanthus *linkage group 13, which are also syntenic with sorghum chromosome 7, are shown in a darker blue. **Panel B**. Circos plot showing centromeric insertion of sorghum chromosome 7 into sorghum chromosome 4 to form *Miscanthus *linkage group 7 (approximate boundaries indicated by arrows). Each line represents an orthologous relationship between a mapped *Miscanthus *marker and its unique counterpart on the *Sorghum bicolor *genome. Both *Miscanthus *linkage groups 7 and 8 have a region corresponding to sorghum chromosome 4, which is inverted with respect to the other markers (dark green arrow and lines). As also shown, *Miscanthus *linkage group 8 is an intact copy of sorghum chromosome 4, and *Miscanthus *linkage group 13 is an intact copy of sorghum chromosome 7.

A simple dot plot (Figure 4A) strikingly reveals complete whole-genome duplication in *M. sinensis *relative to sorghum, with most chromosomes showing near perfect colinearity at the scale of our genetic map. After recognizing this extensive synteny, we oriented and renumbered the *Miscanthus *linkage groups to emphasize this correspondence between *Miscanthus *and sorghum. Every sorghum chromosome exhibits nearly complete marker synteny with a pair of *Miscanthus *linkage groups. Eight sorghum chromosomes are completely duplicated, showing a 1:2 correspondence to *Miscanthus *linkage groups. We infer the whole genome nature of the duplication by the density of colinear markers in sorghum euchromatin, where our gene-biased markers are found. The only evident rearrangement in these chromosomes is a small inversion near the top of Sb4 relative to Ms8 and Ms9. Since Ms8 and Ms9 share the same ordering in this region, this inversion either occurred in the sorghum lineage, or in the stem lineage of *Miscanthus *prior to the tetraploidization event, or is an error in the sorghum genetic map or sequence assembly.

The remaining two sorghum chromosomes, Sb4 and Sb7, are also duplicated over their entire euchromatic spans, but show a more complex pattern of synteny with *Miscanthus*. Ms8 is an intact copy of Sb4, and Ms13 is an intact copy of Sb7. The second copies of these two sorghum chromosomes, however, are fused into the single linkage group Ms7. Ms7 then appears as a copy of Sb7 inserted into the centromeric region of Sb4 (Figure 4B). This single fusion explains the odd base chromosome number of *Miscanthus*. By following the relative orientations of sorghum chromosome arms in *Miscanthus*, we see that this fusion has the characteristic form of a type of insertion previously observed in other grasses [39]. Since all *Miscanthus *species have the same base chromosome number, this fusion presumably occurred in the lineage leading to the last common *Miscanthus *ancestor.

### Mapping C4-PPDK loci in *Miscanthus*

C4 photosynthesis in the Panicoideae (including maize, Saccharinae, millet, switchgrass, *Miscanthus*) is facilitated by a C4-specific form of the pyruvate, phosphate dikinase enzyme (C4-PPDK). Physiological and molecular evidence suggest that altered expression of C4-PPDK may contribute to cold tolerant C4 photosynthesis in *Miscanthus *x *giganteus *[40,41]. The closely related *Sorghum bicolor *has a single C4-PPDK gene located on chromosome 9 [5]. Sequencing of cloned cDNAs from triploid *Miscanthus *x *giganteus *identified five distinct transcripts, including one apparent pseudogene [40], which suggests even greater genetic complexity than three homoeologous C4-PPDK alleles. Based on our observation of whole genome duplication, we reasoned that *M. sinensis *might have an unlinked pair of paralogous C4-PPDK genes. Based on synteny considerations, we expected that these C4-PPDK's would lie on *Miscanthus *LG's, 16 and 17, both of which are syntenic to Sorghum 9.

**Figure 5:**
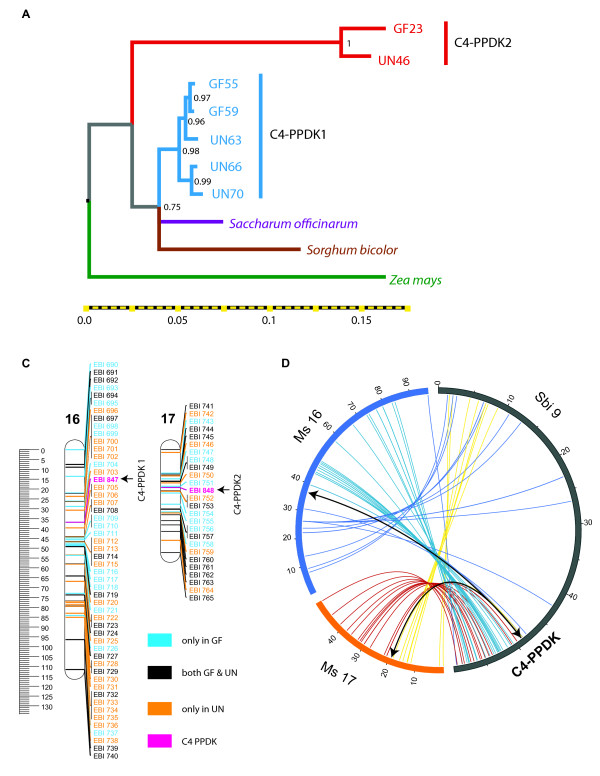
**Mapping PPDK**. **(A) **Phylogenetic tree of representative C4-PPDK sequences showing the clear separation of the two paralogous sequences. **(B) **Placement of the C4-PPDK markers, EBI 847 and EBI 848 (pink) on the linkage map. **(C) **Synteny of corresponding linkage groups (Ms16 and Ms17) to Sorghum chromosome 9. The position of PPDK is illustrated by the black arrows.

To look for C4-PPDK paralogs in *M. sinensis*, and to identify the genetic map position or positions of these gene(s), we designed primers to amplify the third intron of the gene based on the single previously known *Miscanthus *C4-PPDK. Two amplicons were observed in both parents, and both were cloned and sequenced. Cladistic methods identified two distinct paralogs of C4-PPDK (Figure 5A), which we named C4-PPDK1 and C4-PPDK2.

By aligning partial sequences of C4-PPDK in *M. sinensis *with the homologous sequence in *S. bicolor, S. officinarum*, and *Z. mays*, we measured the sequence divergence and phylogenetic relationship between the two *Miscanthus *homoeologs and homologous sequences in related outgroups (Figure 5A). The divergences between Ms C4-PPDK1 and sorghum and sugarcane C4-PPDK are comparable, suggesting that the origin of *Miscanthus *could be contemporaneous with the split between sorghum and sugarcane. Ms C4-PPDK2 branches outside of the Ms C4-PPDK1/sorghum/sugarcane clade, which could indicate that the other parent involved in *Miscanthus *tetraploidy was more divergent. These inferences, however, are weak due to the limited sequence length used in the analysis.

To map the two evident paralogs of C4-PPDK, we designed markers for each gene based on observed intronic sequence variation. Marker EBI 847 is a Cleaved Amplified Polymorphic Sequence (CAPS) marker designed to detect the SNV at position 397 in Additional file 6: Figure S6A, and marker EBI 848 is a sequence length polymorphism (SLP) marker that detects two indels between 1354 bp and 1388 bp (Additional file 6: Figure S6B). Both markers show a 1:1 segregation ratio (Additional file 13: Table S7). EBI 847 maps to *Miscanthus *linkage group Ms16 at 36.8 cM on the integrated map (41.2 cM on the GF maximum likelihood map) while EBI 848 is placed on linkage group Ms17 at 19.2 cM on the integrated map (20.1 cM on the UN maximum likelihood map). *Miscanthus *linkage groups 16 (C4-PPDK1) and 17 (C4-PPDK2) are the homoeologs of *S. bicolor *chromosome 9, which contain sorghum C4-PPDK (Figure 5D). This demonstrates both the utility of our genetic map and sorghum synteny for mapping genes in *Miscanthus*. This is the first documentation of the presence of two paralogous (indeed, homoeologous) C4-PPDKs in *Miscanthus*. The presence of two paralogs provides an opportunity for regulatory divergence and could contribute to the ability of *Miscanthus *to perform cold tolerate photosynthesis.

## Conclusions

All grasses are paleopolyploid by virtue of an ancient whole genome duplication that occurred ~70 million years ago (mya) in a common ancestor of extant Poaceae [42-44]. Many lineages within the grasses have also experienced more recent polyploidization events superimposed on this early event. Here we have shown that *Miscanthus sinensis *is a recent polyploid. Through comparative analysis of our *M. sinensis *genetic map with the *Sorghum bicolor *genome, we account for the base chromosome number x = 19 of the genus *Miscanthus *by a doubling of the ancestral Sacccharinae number x = 10, and a subsequent chromosome fusion. Some taxonomists have included in the *Miscanthus *genus several African accessions that have a base chromosome number of x = 15 (Amalraj and Balasundaram 2006; Hodkinson et al. 1997; 2002) and Himalayan accessions where 2N = 40 (Amalraj and Balasundaram 2006). These may represent ancestral configurations (e.g., 2N = 40), additional karyotypic changes (x = 15), or misclassifications.

Since most common *Miscanthus *species (*M. sinensis, M. sacchariflorus, M. lutarioriparia, M. floridulus*) share the base chromosome number 19, both the genome duplication event and the chromosome fusion likely occurred within the last several million years, at or near the base of the *Saccharinae*. Although we cannot rule out recurrent polyploidizations in the lineages of multiple *Miscanthus *species, a single origin is most parsimonious. Our *M. sinensis *map is consistent with disomic inheritance, without pairing of homoeologous chromosomes despite their limited sequence divergence. The situation in Miscanthus is similar to that found in hexaploid wheat, where closely related species hybridized in allopolyploid fashion, retaining their original chromosomal pairing patterns in a larger genome. Tetraploidization provides the opportunity for a lineage to explore the regulatory and functional diversification of duplicated genes [45-48].

Remarkably, when measured in map units, the *M. sinensis *and *S. bicolor *genetic maps are linearly related, indicating that the inserted repetitive sequence in the *Miscanthus *genome is not recombinogenic (Additional file 14: Figure S5). The total length of the 19 linkage groups of our *M. sinensis *map (~1890 cM) is comparable to the map length of the 10 linkage groups in the *S. bicolor *genome (~1605 cM [32]). Naively, the doubling of chromosome number would be expected to substantially increase the total map length, based on the rule of thumb that each chromosome arm experiences approximately one crossover per meiosis. This suggests that the *Miscanthus *duplication is recent enough that whatever cellular mechanism is responsible for regulating crossover frequency has not had time to adjust to the new karyotype.

**Figure 6:**
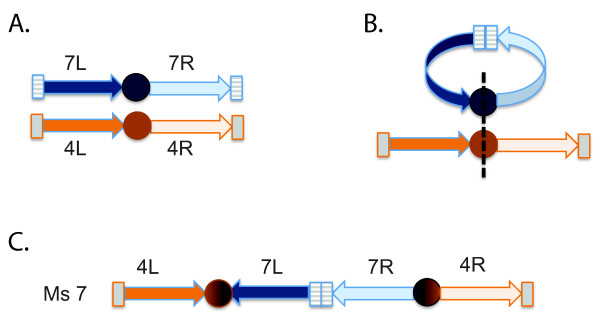
**Mechanism for insertional dysploid reduction of ancestral chromosomes **[36]. The arms of the progenitors of Sb7 and Sb4 are indicated in blue and orange, respectively. Centromeres are shown as solid circles, and telomeres as grey rectangles. **(A) **original configuration. **(B) **intermediate state in which (1) chromosome 7 circularizes, and (2) 7 and 4 recombine at breaks that occur within centromeres. The order of occurrence of circularization and break/recombination are unspecified. **(C) **The resulting order and orientation matches that found in Ms7 (Figure 4B). Note that the original telomeres of 7 lie within the fused chromosome, and are presumably lost. Only one of the two centromeres (shown as mix of orange and blue) survives. An analogous event is proposed to have produced chromosome 4 of *Aegilops tauschii *[36].

It is tempting to speculate that the ensuing chromosome fusion was a critical evolutionary event that established the genus through reproductive isolation of the nascent *Miscanthus *population. Subsequent radiation could then have produced numerous *Miscanthus *species, some of which (e.g., tetraploid *M. sacchariflorus*) underwent additional polyploidization. The nature of these later events is unknown. The ancestral chromosome fusion itself can be understood as arising from the insertion of one chromosome into the centromeric region of another (Figure 6). Insertional fusions have been inferred in other grasses [36], and an insertion with the same orientational properties that we observed has been described for *Aegilops tauschii *chromosome 4D.

The recent and extensive nature of the *Miscanthus *genome duplication, coupled with our use of RNA-seq to discover single nucleotide variant markers, required a careful analysis of segregation patterns in our F1 mapping population to extract *bona fide *allelic polymorphisms from a background of comparable sequence variation that arises from fixed differences between paralogous (and nominally homoeologous) loci. Given the large genome size of *Miscanthus*, deep RNA-seq was an efficient and cost-effective way to identify many single nucleotide variants. Our integration of the resulting single nucleotide polymorphism markers with simple sequence repeat markers confirms the validity of this approach. We took advantage of a new maximum likelihood method for full sib mapping [30] that allows the integration of parental maps. These methods may be useful for rapidly developing markers and maps for other species with complex ploidy.

Since our *M. sinensis *genetic map has good coverage of all 19 linkage groups, and shows limited segregation distortion that is clustered in three regions, we anticipate that it will be useful for further exploration of the *Miscanthus *genome. As a first step in this direction, we used our genetic map and the knowledge that *Miscanthus *is recently duplicated relative to sorghum to discover and map two homoeologous copies of the C4 pyruvate, phosphate dikinase enzyme (C4-PPDK), which appears at the expected syntenic position relative to sorghum C4-PPDK. Whether or not the two C4-PPDK genes have distinct roles is unknown. The ability to separate homoelogous loci suggests that our map could be valuable for both identifying quantitative trait loci in *Miscanthus*, and for marker-assisted breeding improvement of this emerging bioenergy crop.

## Abbreviations

LG: Linkage group. Collection of genetically co-segregating markers that corresponds to a physical chromosome; RAPD: Random amplification of polymorphic DNA. A genotyping method based on annealing of single short primers in configurations that allow for successful PCR amplification; SNVs: Single nucleotide variants. These can occur between true alleles or close paralogs; SNPs: Single nucleotide polymorphisms between alleles. Such segregating SNVs that can be used as genetic markers for mapping; SSRs: Simple Sequence Repeats. PCR-amplified fragments that harbor variable number of short (1-6) bp tandemly-repeated units. The lengths of these tracts are often polymorphic between alleles in a population; SLPs: Sequence Length Polymorphism. PCR-amplified fragments that harbor a difference in size: including SSRs; CAPS: Cleaved Amplified Polymorphic Sequence: PCR-amplified fragments that harbor a polymorphism in a restriction enzyme recognition sequence. The polymorphic state can be detected by digesting the PCR product with the restriction enzyme; UN: *M. sinensis *'Undine': one of the parents of the population used to construct the genetic map; GF: *M. sinensis *'Grosse Fontaine': one of the parents of the population used to construct the genetic map; C4-PPDK: C4-pyruvate: phosphate dikinase.

## Competing interests

The authors declare that they have no competing interests.

## Authors' contributions

KS, SPM, and DSR led the project and oversaw the analysis. WBC made, planted, and maintained the mapping population, led SSR analysis, made regression maps, and mapped C4-PPDK. KS and TM identified SNVs, designed GoldenGate array, analyzed genotyping data, made map, and analyzed sorghum synteny. KG made the doubled haploids under supervision of SJ. AB made RNA for Illumina libraries. KV wrote scripts to analyze GoldenGate data and contributed to figures. LX cloned and analyzed C4-PPDK intronic sequences and designed C4-PPDK markers. MHa, MHu and RM contributed to generation and analysis of genetic map. JJ oversaw development of mapping cross. KS, WBC, and DSR wrote the paper with input from SPM and TM. SPM: requests of materials, DSR communicating paper. All authors reviewed and approved the manuscript.

## Supplementary Material

Additional file 1 **Table S1**. Phenotypes of the *M.sinensis *'Grosse Fontaine' and *M.sinensis *'Undine' parents, as measured in mature plants grown in the greenhouse (Figure 1 panels C). Click here for file

Additional file 2 **Fasta file of the *M.sinensis *RNAseq assembly**. Click here for file

Additional file 3 **Table S3**. GoldenGate OPA containing the probe details for every marker, as provided by Illumina. Click here for file

Additional file 4 **Table S4**. File containing the primer details for all the SSRs. Click here for file

Additional file 5 **Figure S2**. Different amplicon profiles seen in the fragment analysis of SSR markers. The length of the amplicon, in bp, is shown on the horizontal axis and the fluorescence intensity on the vertical axis. Several profiles show "stutter peaks" that are associated with a main peak. These are not counted as distinct marker states. Click here for file

Additional file 6 **Figure S6**. Sequence alignment of two independent regions of *Miscanthus *PPDK paralogs 1 and 2 illustrating indels and SNV used as molecular markers to place C4-PPDK on the linkage map. A) 129 base pairs of sequence from PPDK paralog 1. The G/A single nucleotide polymorphism was converted into the CAPS marker, EBI 847. B) 129 base pairs of PPDK paralog 2 containing indels that were converted into a sequence length polymorphism marker, EBI 848. The oligonucleotide primers, UD3F and UD3R, used to amplify this region are shown. Click here for file

Additional file 7 **Table S2**. RNA sequencing and assembly data table. Click here for file

Additional file 8 **Figure S1**. Distribution of sorghum gene models (top stripe, green), Grosse Fontaine and Undine RNAseq reads (middle stripe, depth in log scale Blue-Orange-Green), and genotyped SNVs (bottom stripe, red) along the ten *Sorghum bicolor *chromosomes. Each chromosome shown to scale (length in Mb shown to left). Click here for file

Additional file 9 **Table S5**. Markers listed in the order of occurrence on the combined maximum likelihood linkage map, with linkage group and map position in cM. Click here for file

Additional file 10 **Figure S3**. Colinearity dot plots of the two mapping methods, maximum likelihood and regression (Kosambi). Click here for file

Additional file 11 **Figure S4**. Colinearity dot plots of the Grosse Fontaine maps versus the Undine maps made using the maximum likelihood (top) and regression algorithms (bottom). Click here for file

Additional file 12 **Table S6**. Genotype scores and marker statistics including segregation distortion for markers and mapping statistics for the integrated map shown in Figure 3. Click here for file

Additional file 13 **Table S7**. Details of revised linkage groups 16 and 17 including two C4-PPDK markers. Click here for file

Additional file 14 **Figure S5**. Linearity of genetic maps for *Sorghum bicolor *and *Miscanthus sinensis*. Markers with unique placement in sorghum were assigned map positions by interpolation relative to the map of Mace et al. 2009 [30]. Click here for file

## References

[B1] D'Hont A Ison D Alix K Roux C Determination of basic chromosome numbers in the genus Saccharum by physical mapping of ribosomal RNA genes Genome 1998 225 221 225

[B2] Sreenivasan TV Ahloowalia BS Heinz D Cytogenetics. Chapter 5. Sugarcane Breeding through breeding 1987 Amsterdam: Elsevier 211 253

[B3] Brandes E Origin, dispersal and use in breeding of the Melanesian garden sugarcane and their derivatives, Saccharum officinarum L Proceedings of the International Society of Sugar Cane Technologists 1956 9 709 750

[B4] D'Hont A Glaszmann JC HD M Sugarcane genome analysis with molecular markers: a first decade of research International Society of Sugar Cane Technologists. Proceedings of the XXIV Congress 2001 2 Brisbane, Australia 556 559

[B5] Paterson AH Bowers JE Bruggmann R Dubchak I Grimwood J Gundlach H Haberer G Hellsten U Mitros T Poliakov A Schmutz J Spannagl M Tang H Wang X Wicker T Bharti AK Chapman J Feltus FA Gowik U Grigoriev IV Lyons E Maher Ca Martis M Narechania A Otillar RP Penning BW Salamov Aa Wang Y Zhang L Carpita NC Freeling M Gingle AR Hash CT Keller B Klein P Kresovich S McCann MC Ming R Peterson DG Mehboob-ur-Rahman Ware D Westhoff P Mayer KFX Messing J Rokhsar DS The Sorghum bicolor genome and the diversification of grasses Nature 2009 457 551 556 10.1038/nature07723 19189423

[B6] Paterson AH Freeling M Tang H Wang X Insights from the comparison of plant genome sequences Annu Rev Plant Biol 2010 61 349 372 10.1146/annurev-arplant-042809-112235 20441528

[B7] Rayburn A Crawford J Rayburn C Juvik J Genome size of three Miscanthus species Plant Mol Biol Rep 2009 27 184 188 10.1007/s11105-008-0070-3

[B8] Price HJ Dillon SL Hodnett G Rooney WL Ross L Johnston JS Genome evolution in the genus Sorghum (Poaceae) Ann Bot 2005 95 219 227 10.1093/aob/mci015 15596469 PMC4246720

[B9] Swaminathan K Alabady MS Varala K De Paoli E Ho I Rokhsar DS Arumuganathan AK Ming R Green PJ Meyers BC Moose SP Hudson ME Genomic and small RNA sequencing of Miscanthus x giganteus shows the utility of sorghum as a reference genome sequence for Andropogoneae grasses Genome Biol 2010 11 R12 10.1186/gb-2010-11-2-r12 20128909 PMC2872872

[B10] Swigonová Z Lai J Ma J Ramakrishna W Llaca V Bennetzen JL Messing J Close split of sorghum and maize genome progenitors Genome Res 2004 14 1916 1923 10.1101/gr.2332504 15466289 PMC524415

[B11] Draper J Mur L Jenkins G Brachypodium distachyon. A new model system for functional genomics in grasses Plant 2001 127 1539 1555 PMC133562 11743099

[B12] Burner D Cytogenetic analyses of sugarcane relatives (Andropogoneae: Saccharinae) Euphytica 1991 54 125 133 10.1007/BF00145639

[B13] Lafferty J Lelley T Cytogenetic Studies of Different Miscanthus Species with Potential for Agricultural Use Plant Breeding 1994 113 246 249 10.1111/j.1439-0523.1994.tb00730.x

[B14] Linde-Laursen I Cytogenetic analysis of Miscanthus "Giganteus", an interspecific hybrid Hereditas 1993 119 297 300

[B15] Heaton Ea Dohleman FG Long SP Meeting US biofuel goals with less land: the potential of Miscanthus Glob Chang Biol 2008 14 2000 2014 10.1111/j.1365-2486.2008.01662.x

[B16] Hodkinson TR Characterization of a Genetic Resource Collection for Miscanthus (Saccharinae, Andropogoneae, Poaceae) using AFLP and ISSR PCR Ann Bot 2002 89 627 636 10.1093/aob/mcf091 12099538 PMC4233896

[B17] Atienza G Satovic Z Petersen K Dolstra O Martín A Preliminary genetic linkage map of Miscanthus sinensis with RAPD markers Theor Appl Genet 2002 105 946 952 10.1007/s00122-002-0956-7 12582920

[B18] Ming R Liu SC Lin YR da Silva J Wilson W Braga D van Deynze A Wenslaff TF Wu KK Moore PH Burnquist W Sorrells ME Irvine JE Paterson AH Detailed alignment of saccharum and sorghum chromosomes: comparative organization of closely related diploid and polyploid genomes Genetics 1998 150 1663 1682 9832541 10.1093/genetics/150.4.1663 PMC1460436

[B19] Okada M Lanzatella C Saha MC Bouton J Wu R Tobias CM Complete switchgrass genetic maps reveal subgenome collinearity, preferential pairing and multilocus interactions Genetics 2010 185 745 760 10.1534/genetics.110.113910 20407132 PMC2907199

[B20] Chang S Puryear J A simple and efficient method for isolating RNA from pine trees Plant Mol Biol Rep 1993 11 113 116 10.1007/BF02670468

[B21] Birol I Jackman SD Nielsen CB Qian JQ Varhol R Stazyk G Morin RD Zhao Y Hirst M Schein JE Horsman DE Connors JM Gascoyne RD Marra MA Jones SJM De novo transcriptome assembly with ABySS Bioinformatics (Oxford, England) 2009 25 2872 2877 10.1093/bioinformatics/btp367 19528083

[B22] Langmead B Trapnell C Pop M Salzberg SL Ultrafast and memory-efficient alignment of short DNA sequences to the human genome Genome Biol 2009 10 R25 10.1186/gb-2009-10-3-r25 19261174 PMC2690996

[B23] Langmead B Aligning short sequencing reads with Bowtie Curr Protoc Bioinformatics 2010 11 1 24 10.1002/0471250953.bi1107s32 PMC3010897 21154709

[B24] Li H Durbin R Fast and accurate short read alignment with Burrows-Wheeler transform Bioinformatics (Oxford, England) 2009 25 1754 1760 10.1093/bioinformatics/btp324 PMC2705234 19451168

[B25] Li H Durbin R Fast and accurate long-read alignment with Burrows-Wheeler transform Bioinformatics (Oxford, England) 2010 26 589 595 10.1093/bioinformatics/btp698 PMC2828108 20080505

[B26] Li H Handsaker B Wysoker A Fennell T Ruan J Homer N Marth G Abecasis G Durbin R The Sequence Alignment/Map format and SAMtools Bioinformatics (Oxford, England) 2009 25 2078 2079 10.1093/bioinformatics/btp352 PMC2723002 19505943

[B27] Koboldt DC Chen K Wylie T Larson DE McLellan MD Mardis ER Weinstock GM Wilson RK Ding L VarScan: variant detection in massively parallel sequencing of individual and pooled samples Bioinformatics (Oxford, England) 2009 25 2283 2285 10.1093/bioinformatics/btp373 PMC2734323 19542151

[B28] Kent WJ BLAT--The BLAST-Like Alignment Tool Genome Res 2002 12 656 664 11932250 10.1101/gr.229202 PMC187518

[B29] James BT Chen C Rudolph A Swaminathan K Murray JE Na J-K Spence AK Smith B Hudson ME Moose SP Ming R Development of microsatellite markers in autopolyploid sugarcane and comparative analysis of conserved microsatellites in sorghum and sugarcane Molecular Breeding 2011

[B30] VAN Ooijen JW Multipoint maximum likelihood mapping in a full-sib family of an outbreeding species Genet Res 2011 93 343 349 10.1017/S0016672311000279 21878144

[B31] Camacho C Coulouris G Avagyan V Ma N Papadopoulos J Bealer K Madden TL BLAST+: architecture and applications BMC Bioinforma 2009 10 421 10.1186/1471-2105-10-421 PMC2803857 20003500

[B32] Mace ES Rami J-F Bouchet S Klein PE Klein RR Kilian A Wenzl P Xia L Halloran K Jordan DR A consensus genetic map of sorghum that integrates multiple component maps and high-throughput Diversity Array Technology (DArT) markers BMC Plant Biol 2009 9 13 10.1186/1471-2229-9-13 19171067 PMC2671505

[B33] Edgar RC MUSCLE: multiple sequence alignment with high accuracy and high throughput Nucleic Acids Res 2004 32 1792 1797 10.1093/nar/gkh340 15034147 PMC390337

[B34] Castresana J Selection of conserved blocks from multiple alignments for their use in phylogenetic analysis Mol Biol Evol 2000 17 540 552 10.1093/oxfordjournals.molbev.a026334 10742046

[B35] Huelsenbeck J Ronquist F MRBAYES: Bayesian inference of phylogenetic trees Bioinformatics 2001 17 754 755 10.1093/bioinformatics/17.8.754 11524383

[B36] Cordeiro GM Casu R McIntyre CL Manners JM Henry RJ Microsatellite markers from sugarcane (Saccharum spp.) ESTs cross transferable to erianthus and sorghum Plant science: an international journal of experimental plant biology 2001 160 1115 1123 11337068 10.1016/s0168-9452(01)00365-x

[B37] Knapik EW Goodman A Atkinson OS Roberts CT Shiozawa M Sim CU Weksler-Zangen S Trolliet MR Futrell C Innes BA Koike G McLaughlin MG Pierre L Simon JS Vilallonga E Roy M Chiang PW Fishman MC Driever W Jacob HJ A reference cross DNA panel for zebrafish (Danio rerio) anchored with simple sequence length polymorphisms Development 1996 123 451 460 9007262 10.1242/dev.123.1.451

[B38] Chakravarti A Lasher LK Reefer JE A maximum likelihood method for estimating genome length using genetic linkage data Genetics 1991 128 175 182 2060775 10.1093/genetics/128.1.175 PMC1204446

[B39] Luo MC Deal KR Akhunov ED Akhunova AR Anderson OD Anderson JA Blake N Clegg MT Coleman-Derr D Conley EJ Crossman CC Dubcovsky J Gill BS Gu YQ Hadam J Heo HY Huo N Lazo G Ma Y Matthews DE McGuire PE Morrell PL Qualset CO Renfro J Tabanao D Talbert LE Tian C Toleno DM Warburton ML You FM Zhang W Dvorak J Genome comparisons reveal a dominant mechanism of chromosome number reduction in grasses and accelerated genome evolution in Triticeae Proc Natl Acad Sci USA 2009 106 15780 15785 10.1073/pnas.0908195106 19717446 PMC2747195

[B40] Naidu S Moose S Al-Shoaibi A Cold tolerance of C4 photosynthesis in Miscanthus x giganteus: adaptation in amounts and sequence of C4 photosynthetic enzymes Plant Physiol 2003 132 1688 1697 10.1104/pp.103.021790 12857847 PMC167105

[B41] Wang D Portis AR Moose SP Long SP Cool C4 photosynthesis: pyruvate Pi dikinase expression and activity corresponds to the exceptional cold tolerance of carbon assimilation in Miscanthus x giganteus Plant Physiol 2008 148 557 567 10.1104/pp.108.120709 18539777 PMC2528129

[B42] Thiel T Graner A Waugh R Grosse I Close TJ Stein N Evidence and evolutionary analysis of ancient whole-genome duplication in barley predating the divergence from rice BMC Evol Biol 2009 9 209 10.1186/1471-2148-9-209 19698139 PMC2746218

[B43] Salse J Chagué V Bolot S Magdelenat G Huneau C Pont C Belcram H Couloux A Gardais S Evrard A Segurens B Charles M Ravel C Samain S Charmet G Boudet N Chalhoub B New insights into the origin of the B genome of hexaploid wheat: evolutionary relationships at the SPA genomic region with the S genome of the diploid relative Aegilops speltoides BMC Genomics 2008 9 555 10.1186/1471-2164-9-555 19032732 PMC2612700

[B44] Bolot S Abrouk M Masood-Quraishi U Stein N Messing J Feuillet C Salse J The "inner circle" of the cereal genomes Curr Opin Plant Biol 2009 12 119 125 10.1016/j.pbi.2008.10.011 19095493

[B45] Otto SP The evolutionary consequences of polyploidy Cell 2007 131 452 462 10.1016/j.cell.2007.10.022 17981114

[B46] Adams KL Wendel JF Polyploidy and genome evolution in plants Curr Opin Plant Biol 2005 8 135 141 10.1016/j.pbi.2005.01.001 15752992

[B47] Adams KL Percifield R Wendel JF Organ-specific silencing of duplicated genes in a newly synthesized cotton allotetraploid Genetics 2004 168 2217 2226 10.1534/genetics.104.033522 15371349 PMC1448729

[B48] Feldman M Levy AA Genome evolution in allopolyploid wheat-a revolutionary reprogramming followed by gradual changes J Genet Genomics 2009 36 511 518 10.1016/S1673-8527(08)60142-3 19782952

