# Supplementary material for: A framework genetic map for Miscanthus sinensis from RNAseq-based markers shows recent tetraploidy
Source: BMC Genomics. 2012 Apr 24;13:142. doi: 10.1186/1471-2164-13-142 (PMC3355032; doi:10.1186/1471-2164-13-142)
Supplement: Additional file 1 — Table S1. Phenotypes of the M.sinensis 'Grosse Fontaine' and M.sinensis 'Undine' parents, as measured in mature plants grown in the greenhouse (Figure 1 panels C). [file 1471-2164-13-142-S1.DOCX]

| *Miscanthus sinensis* | Grosse Fontaine | Undine |
| --- | --- | --- |
| Height * | 211.0 +/- 27.4 cm | 166.4 +/- 3.8 cm |
| Stem diameter* | 0.63 +/- 0.03 cm | 0.48 +/- 0.02 cm |
| Flowering time* | Mid-late | Early |
| DNA content/cell | 5.17 +/- 0.05 pg | 5.36 +/- 0.20 pg |
| Seeds collected as female parent | <700 | >10,000 |
| Germination rate | 94.1% | 81.2% |
| Number of seedlings | 542 | 1,345 |

**Additional Table 1.** Summary of characteristics of the two parents of our mapping cross, based on one season of growth in greenhouse as described in Methods.

* These measurements are from greenhouse condition with maximum growth (full development).
