# Supplementary figures and images for: A framework genetic map for Miscanthus sinensis from RNAseq-based markers shows recent tetraploidy
Source: BMC Genomics. 2012 Apr 24;13:142. doi: 10.1186/1471-2164-13-142 (PMC3355032; doi:10.1186/1471-2164-13-142)

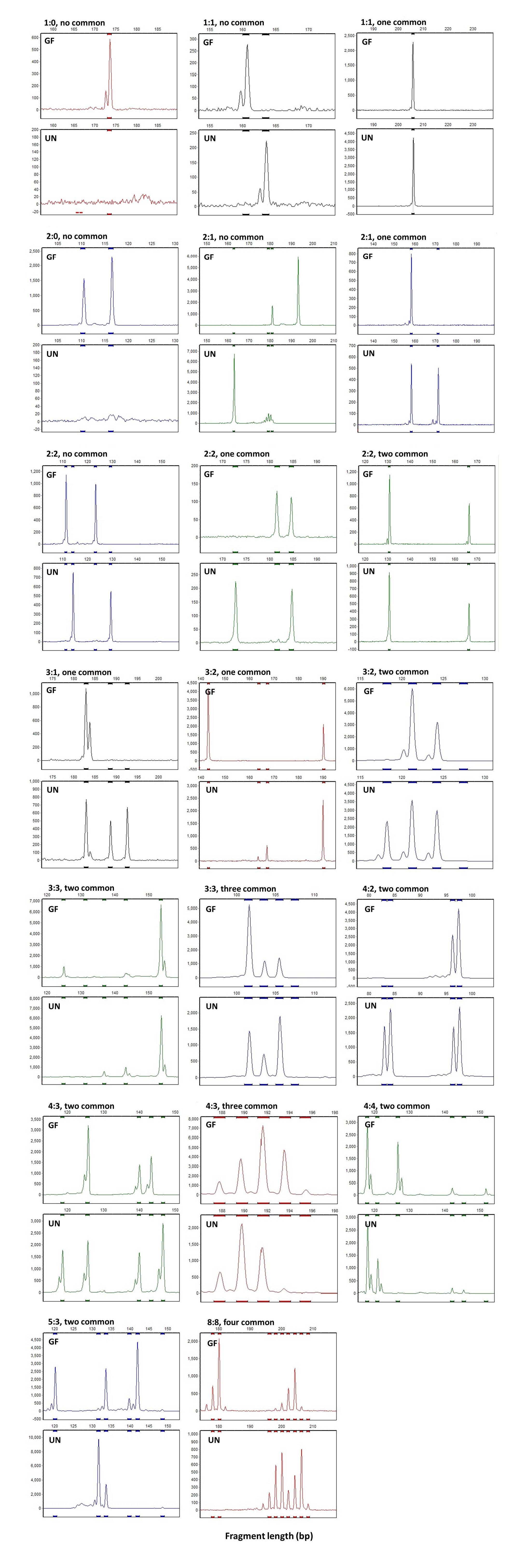

Supplement: Additional file 5 — Figure S2. Different amplicon profiles seen in the fragment analysis of SSR markers. The length of the amplicon, in bp, is shown on the horizontal axis and the fluorescence intensity on the vertical axis. Several profiles show "stutter peaks" that are associated with a main peak. These are not counted as distinct marker states. [file 1471-2164-13-142-S5.JPEG]

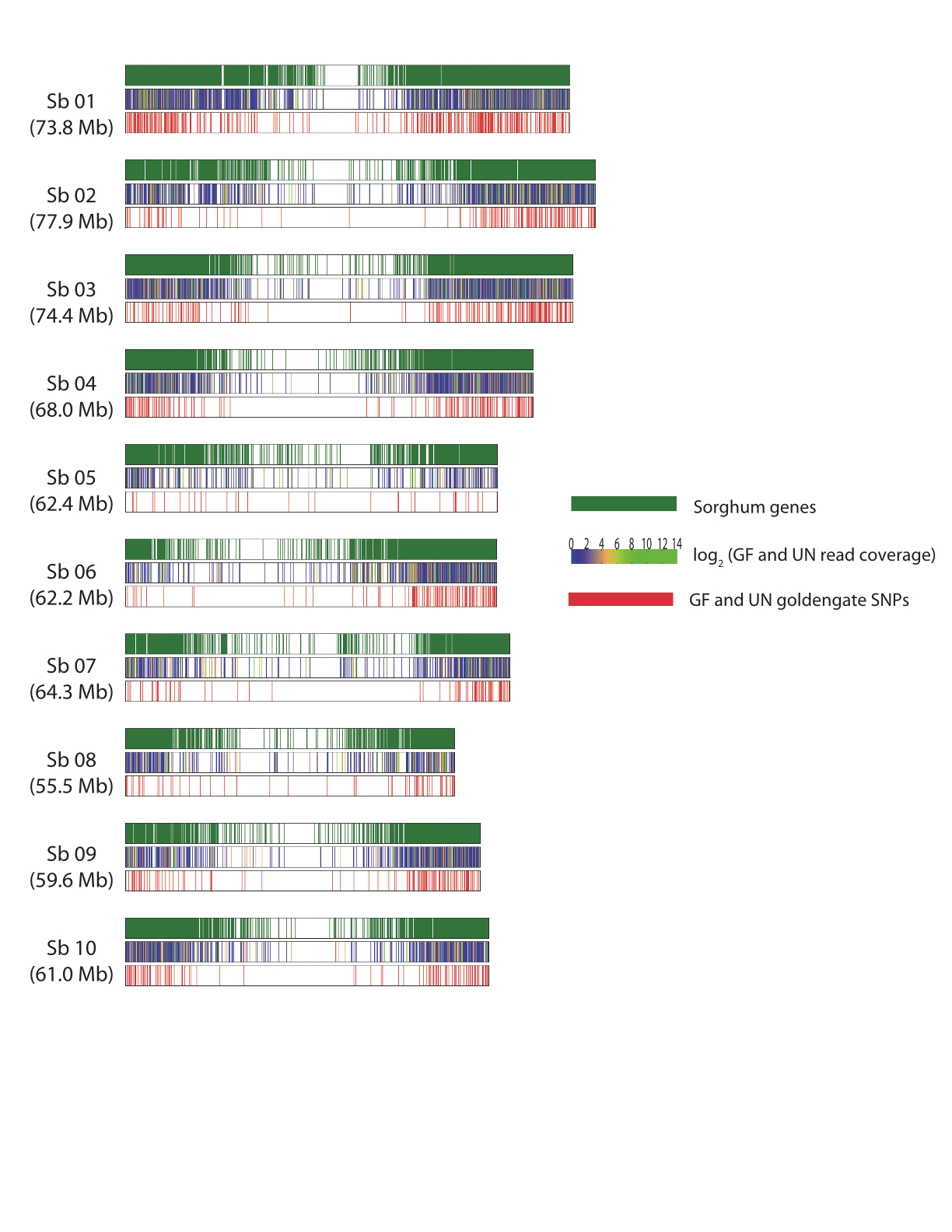

Supplement: Additional file 8 — Figure S1. Distribution of sorghum gene models (top stripe, green), Grosse Fontaine and Undine RNAseq reads (middle stripe, depth in log scale Blue-Orange-Green), and genotyped SNVs (bottom stripe, red) along the ten Sorghum bicolor chromosomes. Each chromosome shown to scale (length in Mb shown to left). [file 1471-2164-13-142-S8.JPEG]

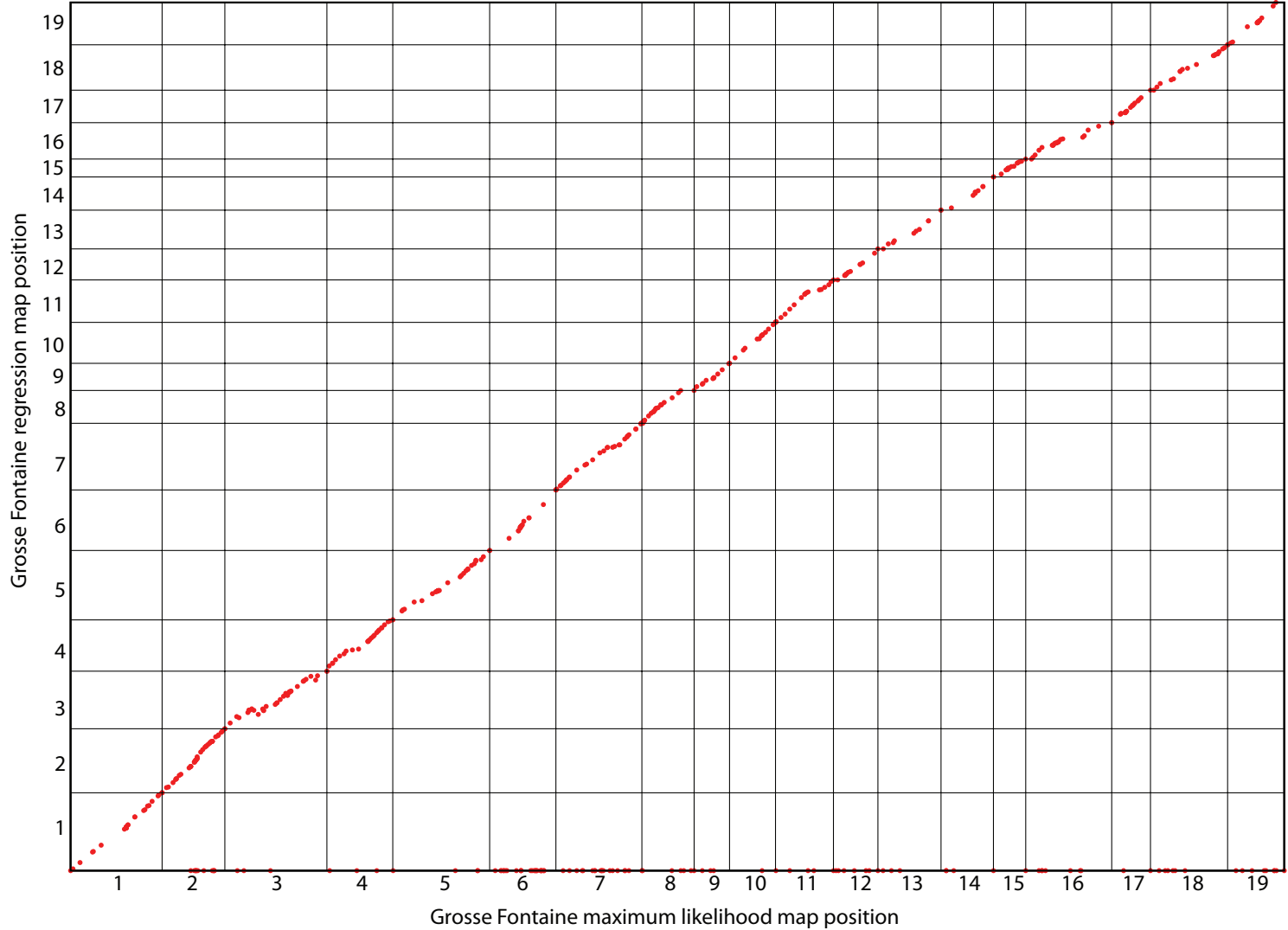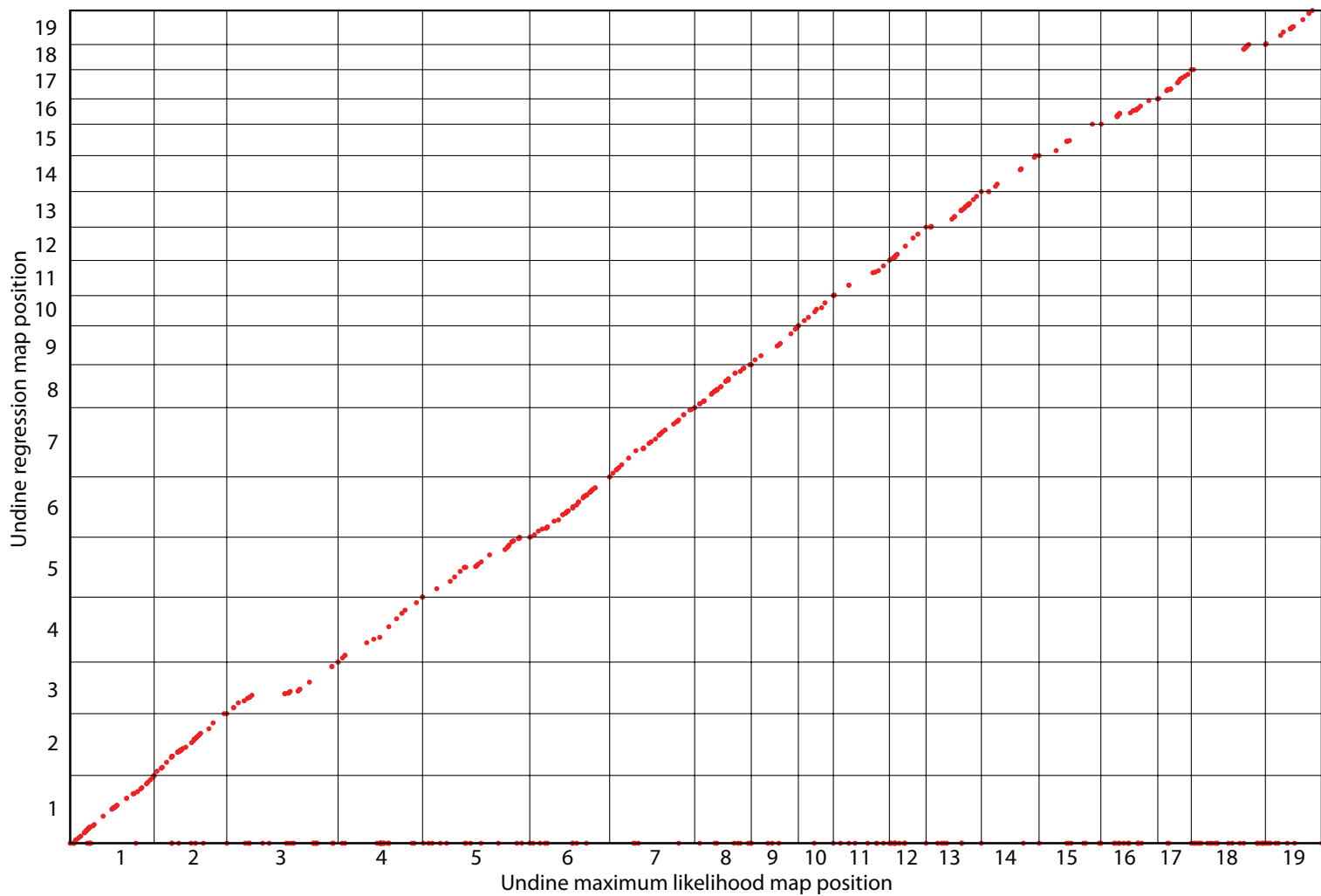

Supplement: Additional file 10 — Figure S3. Colinearity dot plots of the two mapping methods, maximum likelihood and regression (Kosambi). [file 1471-2164-13-142-S10.PDF]

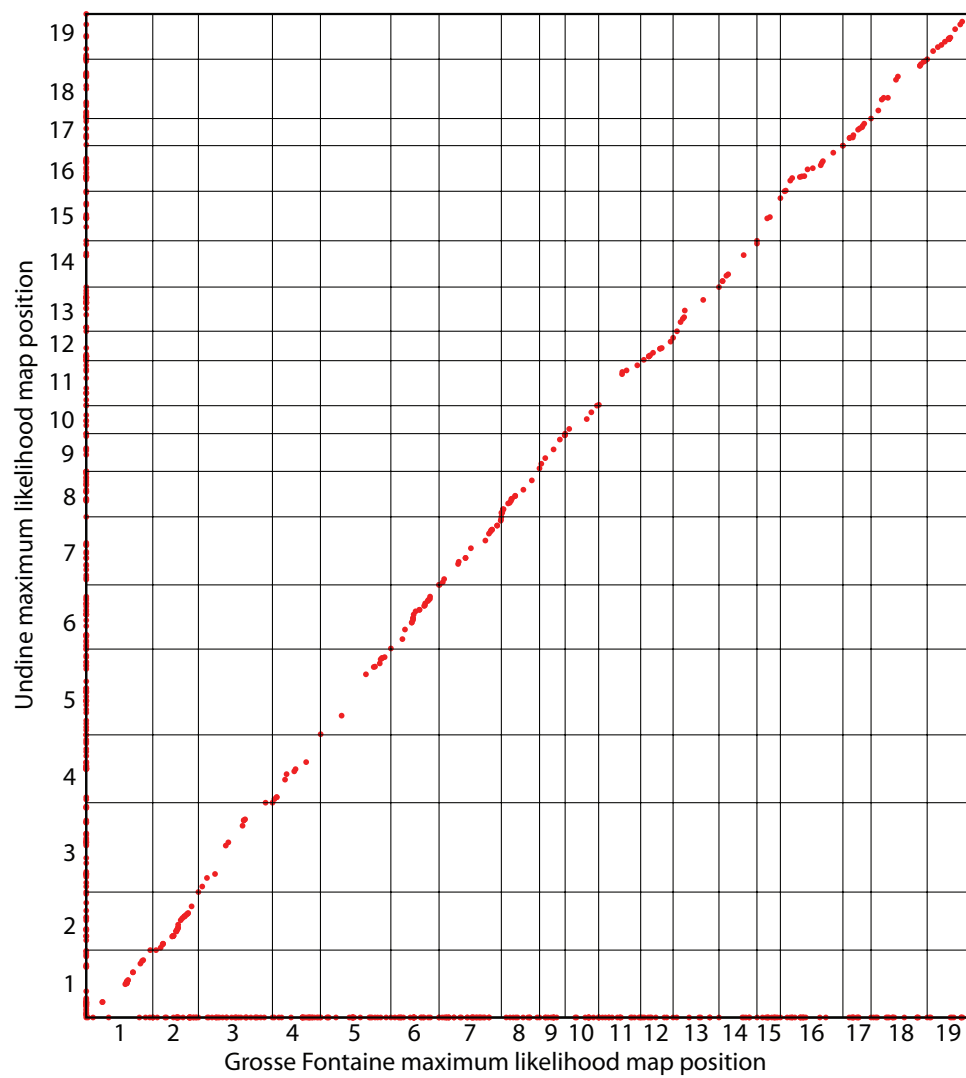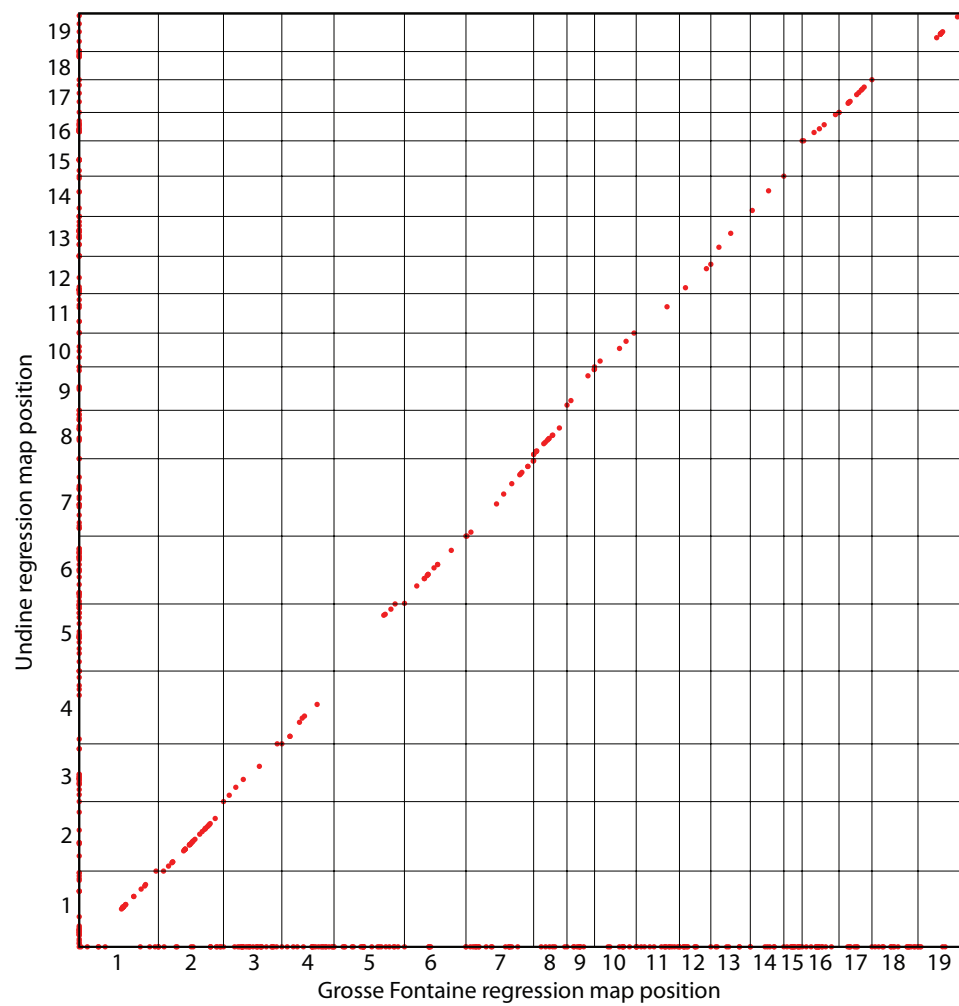

Supplement: Additional file 11 — Figure S4. Colinearity dot plots of the Grosse Fontaine maps versus the Undine maps made using the maximum likelihood (top) and regression algorithms (bottom). [file 1471-2164-13-142-S11.PDF]

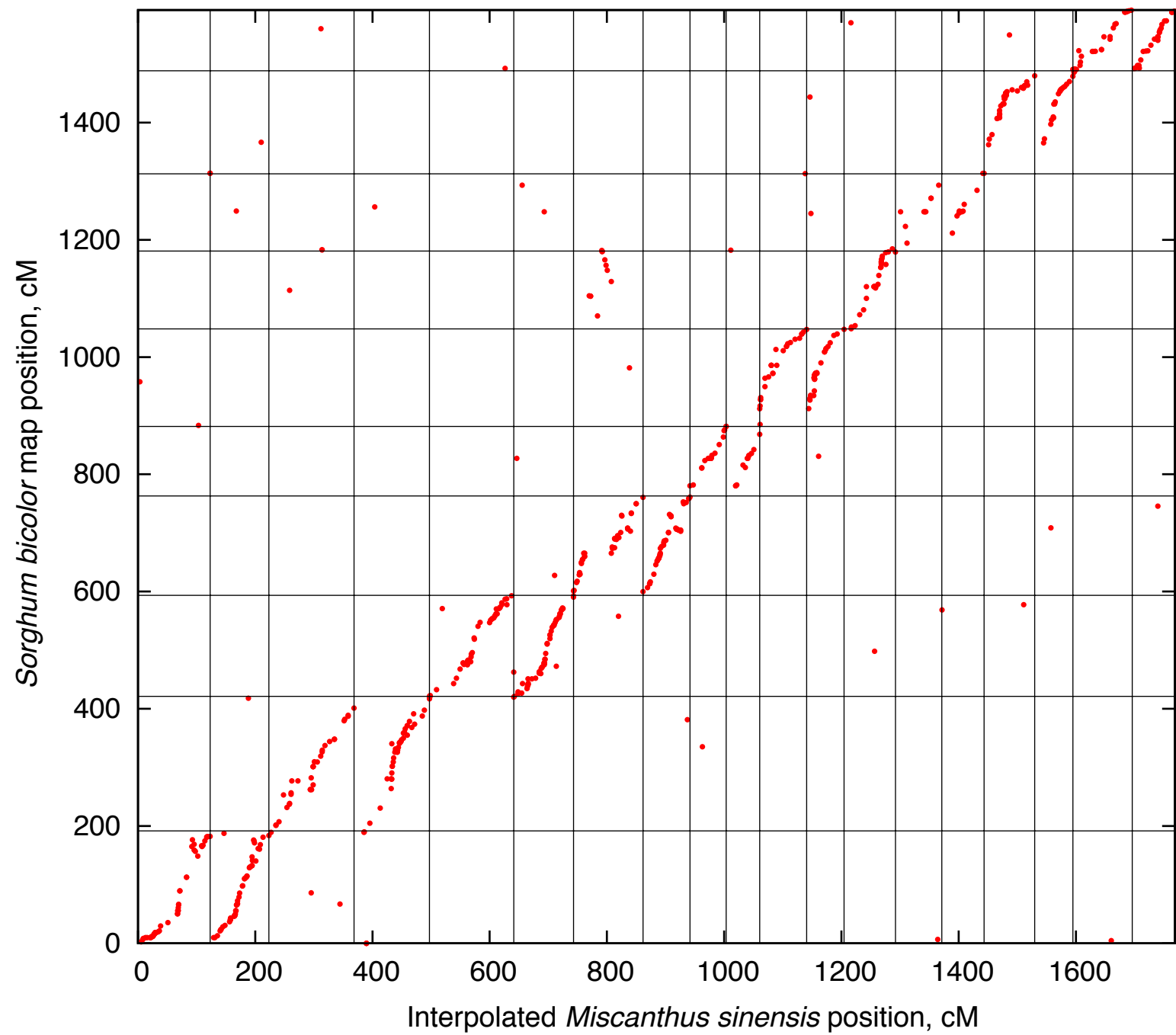

Supplement: Additional file 14 — Figure S5. Linearity of genetic maps for Sorghum bicolor and Miscanthus sinensis. Markers with unique placement in sorghum were assigned map positions by interpolation relative to the map of Mace et al. 2009 [30]. [file 1471-2164-13-142-S14.PDF]
