# Supplementary material for: A framework genetic map for Miscanthus sinensis from RNAseq-based markers shows recent tetraploidy
Source: BMC Genomics. 2012 Apr 24;13:142. doi: 10.1186/1471-2164-13-142 (PMC3355032; doi:10.1186/1471-2164-13-142)

A. C4-PPDK-1 marker

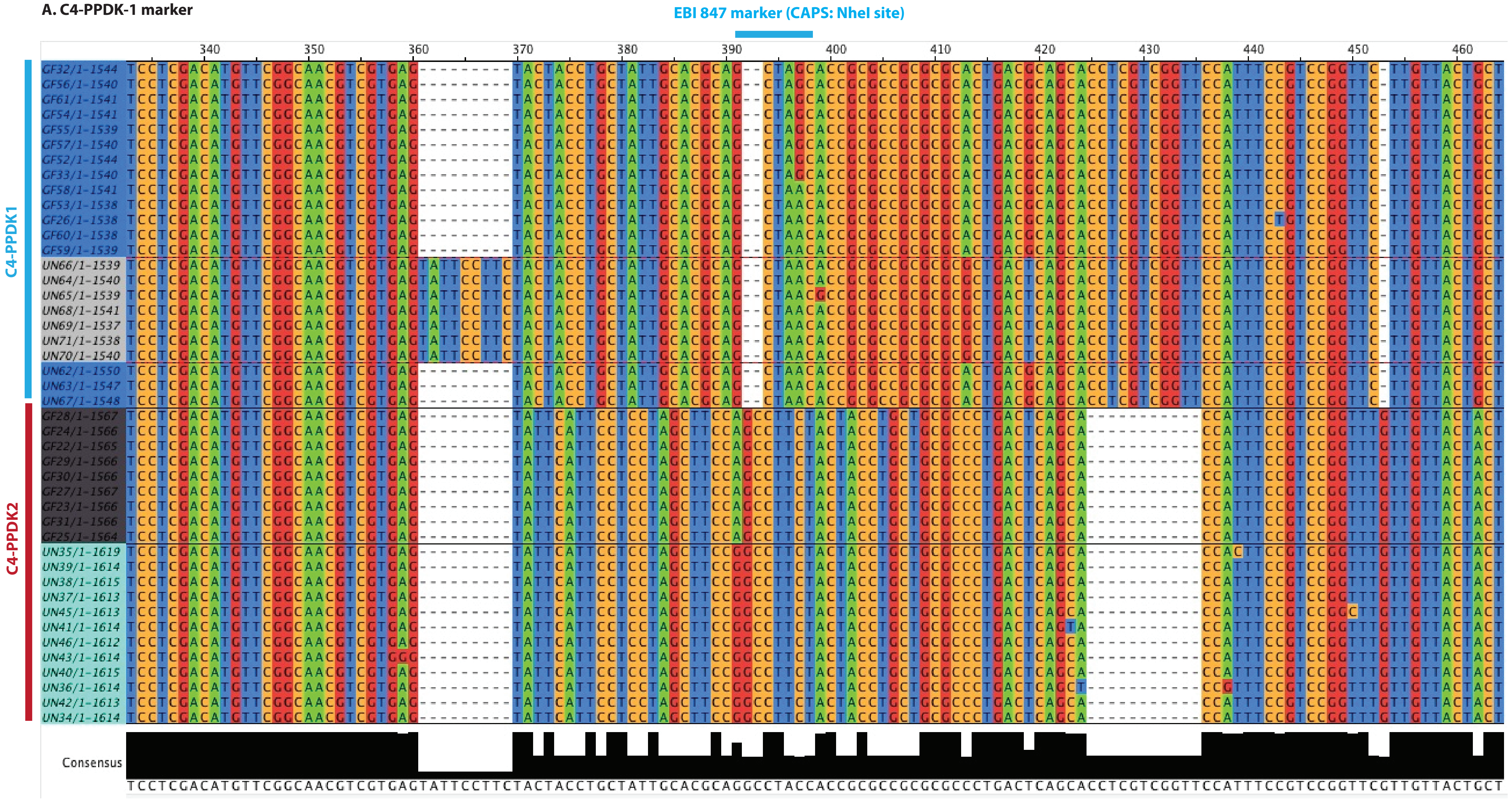

B. C4-PPDK-2 marker

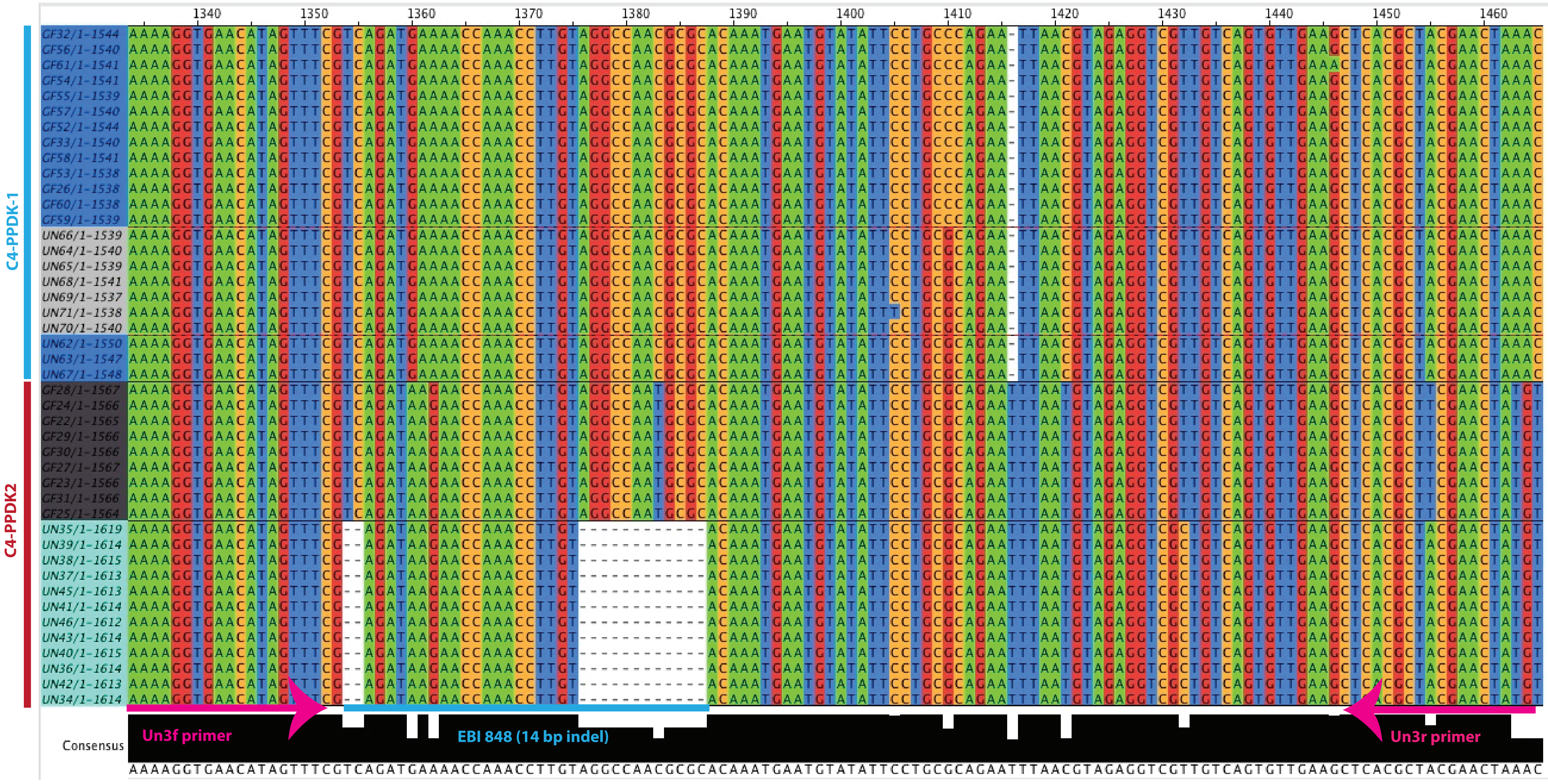

Supplement: Additional file 6 — Figure S6. Sequence alignment of two independent regions of Miscanthus PPDK paralogs 1 and 2 illustrating indels and SNV used as molecular markers to place C4-PPDK on the linkage map. A) 129 base pairs of sequence from PPDK paralog 1. The G/A single nucleotide polymorphism was converted into the CAPS marker, EBI 847. B) 129 base pairs of PPDK paralog 2 containing indels that were converted into a sequence length polymorphism marker, EBI 848. The oligonucleotide primers, UD3F and UD3R, used to amplify this region are shown. [file 1471-2164-13-142-S6.PDF]
