# Supplementary material for: A framework genetic map for Miscanthus sinensis from RNAseq-based markers shows recent tetraploidy
Source: BMC Genomics. 2012 Apr 24;13:142. doi: 10.1186/1471-2164-13-142 (PMC3355032; doi:10.1186/1471-2164-13-142)
Supplement: Additional file 7 — Table S2. RNA sequencing and assembly data table. [file 1471-2164-13-142-S7.DOCX]

| *Miscanthus* *sinensis* Accession | Runs | total no. of reads (80bp) | Total Gbp sequenced | No. of contigs >100 bp | N50  (bp) | No. of contigs >= N50 |
| --- | --- | --- | --- | --- | --- | --- |
| 'Grosse Fontaine' | 3 paired-end runs | 69.9 M | 11.2 | 26,798 | 919 | 5,526 |
| 'Undine' | 2 paired-end and 1 single-ended run | 73.7 M | 10.2 | 17,669 | 889 | 3,815 |

**Additional Table 2**: RNA sequencing and assembly data table.

Note: The assembly will be provided as a fasta supplementary file and the raw reads uploaded to the Genbank Short Read Archive upon acceptance of the manuscript.
